# The Plasma Membrane may Serve as a Drug Depot to Drive the Extreme Potency of Fentanyl

**DOI:** 10.1101/2025.07.22.666146

**Published:** 2025-07-26

**Authors:** Joseph Clayton, George J. Farmer, Jackie Glenn, Shailesh N. Mistry, J. Robert Lane, Lei Shi, Lidiya Stavitskaya, Meritxell Canals, Jana Shen

**Affiliations:** 1 Department of Pharmaceutical Sciences, University of Maryland School of Pharmacy, Baltimore, MD 21201, U.S.A.; 2 Division of Applied Regulatory Science, Office of Clinical Pharmacology, Office of Translational Sciences, Center for Drug Evaluation and Research, United States Food and Drug Administration, Silver Spring, MD 20993, U.S.A.; 3 Division of Biomolecular Science and Medicinal Chemistry, School of Pharmacy, University of Nottingham Biodiscovery Institute, University Park, Nottingham, UK; 4 Centre of Membrane Proteins and Receptors, Universities of Nottingham and Birmingham, Midlands, UK; 5 Division of Physiology, Pharmacology and Neuroscience, School of Life Sciences, University of Nottingham, Queens Medical Centre, Nottingham, UK; 6 Computational Chemistry and Molecular Biophysics Section, National Institutes on Drug Abuse - Intramural Research Pr

## Abstract

The drug overdose crisis in the United States is driven largely by the ultrapotent synthetic (UPS) opioid fentanyl; however, fentanyl’s extreme potency is poorly understood. Here we used state-of-the-art molecular dynamics simulations and experiments to test a hypothesis that fentanyl’s extreme potency is driven by its ability to partition into the plasma membrane, creating a drug reservoir near the receptor. The estimated effective permeability of fentanyl at pH 7.5 is on the order of 10^−7^ cm/s – at least two orders of magnitude faster than morphine. In contrast, isotonitazene (a newly emerged UPS opioid) and naloxone (an antagonist) effectively do not partition into the membrane under the same conditions. The simulations captured the proton-coupled permeation processes, challenging the long-standing pH-partition hypothesis. Subsequent reporter cell line experiments demonstrated that cells exposed to fentanyl, but not morphine, reactivated the receptor even after washout and addition of naloxone. Immobilized affinity membrane chromatography confirmed that fentanyl has significantly higher phospholipid affinity than morphine. Our findings strongly support the drug depot hypothesis and highlight the importance of membrane-dependent opioid pharmacology for understanding toxicity and guiding the design of more effective antagonists. The simulation methodology enables detailed analysis of membrane permeation by ionizable inhibitors, supporting ADME optimization in drug development.

The opioid-related deaths in the United States have increased sharply over the last decade. A new height was reached in 2023, with over 80,000 overdose deaths, among which 90% involved fentanyl (1), which is an ultrapotent synthetic (UPS) opioid exhibiting distinct pharmacology compared to the natural opiate morphine. Comparison of cryogenic electron microscopy (cryoEM) structures of fentanyl- and morphine-bound *μ*OR (2) suggests that fentanyl’s increased potency may result from the additional interactions with the receptor, particularly between its phenylethyl group and a minor pocket in *μ*OR (3). These unique receptor interactions likely contribute to the ~10-fold higher in vitro functional potency of fentanyl compared to morphine (4); however, they cannot explain the drastically greater analgesic potency of fentanyl, which is 50–400 times that of morphine (5). In contrast, recent experiments using whole-cell patch-clamp electrophysiology and signaling assays found that fentanyl, but not morphine, can reactivate *μ*OR after washout, implying membrane retention (6). This lends support to an alternative hypothesis: fentanyl partitions into the plasma membrane, forming a local “drug depot” that may enhance binding kinetics and/or enable lipid-mediated receptor access (7). The hypothesis is consistent with the exceptional lipophilicity of fentanyl (5), as reflected by its octanol-water partition coefficient, which is over 700 fold higher than that of morphine (8) despite their similar solution p*K*_a_ values – a discrepancy that remains poorly understood.

Motivated by the unresolved questions surrounding fentanyl’s extreme potency and lipophilicity and to test the “drug depot” hypothesis, we set out to investigate the membrane permeation properties of three *μ*OR agonist (fentanyl, morphine, and isotonitazene) and the *μ*OR antagonist and opioid reversal agent naloxone (Fig. 1) using state-of-the-art molecular dynamics (MD) simulations and experiments. Compared to fentanyl, isotonitazene is a new ultrapotent synthetic opioid from the 2-benzylbenzimidazole family, which was first identified around 2019 in the Midwest of the USA and subsequently classified as a Schedule I substance by the Drug Enforcement Agency (DEA). To allow direct simulation of membrane permeation processes of titratable molecules with full atomic detail, we integrated the GPU-accelerated particle-mesh Ewald continuous constant pH MD (CpHMD) (9, 10), which captures proton-coupled conformational dynamics, with the weighted-ensemble (WE) protocol (11, 12), which accelerates sampling of rare events such as drug permeation (13). Traditional theoretical studies of passive membrane permeation by small molecules have typically relied on calculating free energy profiles (potential of mean force) along the membrane normal using umbrella sampling (14-16) or other biased sampling protocols such as metadynamics (14,17). In contrast, we report, to the best of our knowledge, the first atomic-level simulations that account for full conformational and protonation flexibility of drugs interacting with the membrane, offering a more realistic and dynamic representation of the permeation process. Additionally, the opioids examined here are significantly larger than the molecules studied by the previous simulation work (13-17). Findings from our simulations and experiments offer compelling support for the “drug depot” hypothesis and an atomically detailed explanation for fentanyl’s exceptional lipophilicity. More broadly, proton-coupled weighted-ensemble MD offers a powerful approach for investigating the membrane permeation of ionizable drugs—a common yet poorly understood aspect of drug pharmacokinetics.

## Results

### Fentanyl partitions into the membrane orders of magnitude faster than other opioids.

**Figure 1:**
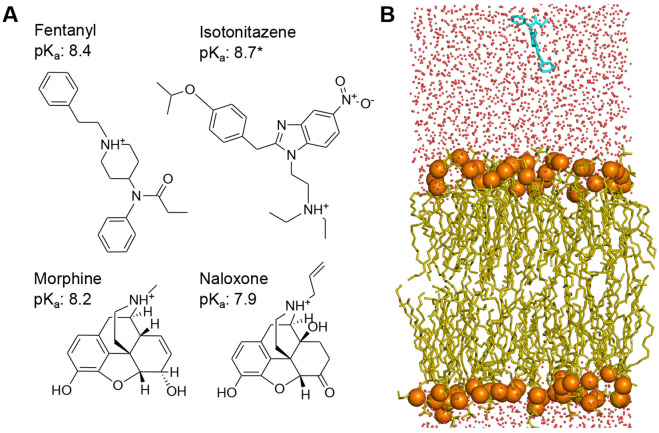
Chemical structures of fentanyl, morphine, isotonitazene, naloxone, and the simulation setup. **A.** Chemical structures and experimental solution p*K*_a_ values of fentanyl (33), isotonitazene (approximated by that of dimethyltryptamine (34)), morphine, (35) and naloxone (36). **B.** A snapshot of fentanyl (cyan) approaching the model lipid bilayer comprised of POPC, POPE, and cholesterol in a 5:5:1 ratio. Water molecules are represented by red dots, and the lipid phosphorous atoms are shown as orange spheres.

The membrane permeation simulations at 1 bar, 300 K, and solution pH 7.5 were conducted for three opioid agonists (fentanyl, morphine, and isotonitazene) and one antagonist (naloxone) using the PME-CpHMD module (10) in Amber24 (18). In order to accelerate the sampling of rare events (i.e., membrane permeation), the weighted ensemble method (11) implemented in WESTPA 2.0 (12) was employed, with the progress coordinate defined as the permeant center of mass (COM) *z*-position relative to that of the lipid bilayer. The simulations were conducted under steady-state conditions and initiated with the permeant placed approximately 10 Å away from a fully solvated lipid bilayer composed of palmitoyloleoylphosphatidylcholine (POPC), palmitoyloleoylphosphatidylethanolamine (POPE) and cholesterol in a 5:5:1 ratio (Fig. 1B and SI Fig. S1). All molecules primarily adopt the charged (protonated amine) state in solution (SI Fig. S2). To confirm our findings, a second set of simulations was conducted for all molecules with a modified WE protocol, where additional WE bins were added near the upper leaflet-water interface to further enhance sampling of the membrane partitioning events. Thus, our discussion will focus on this second of simulations; unless otherwise noted, results from the first set are given in parentheses.

The simulations demonstrate that the membrane permeation rate follows the order: fentanyl > morphine > isotonitazene > naloxone. Based on the probability flux (13), we estimated the mean first passage time (MFPT), which is the average time for the permeant to pass the membrane for the first time. The estimated MFPT of fentanyl is 10 (4.6) s, while those of morphine, isotonitazene, and naloxone are orders of magnitude larger, at 9.8 × 10^2^ (8.8 × 10^5^) s, 3.9× 10^12^ (3.0 × 10^9^) s, and 1.8× 10^30^ s, respectively (Table 1). Note, the MFPT of naloxone could not be estimated in the first trial, as it did not permeate the lower leaflet within the simulation time (SI Fig. S3). These data suggest that: 1) fentanyl is the most capable of partitioning into the membrane; 2) morphine can partition into the membrane, albeit much more slowly than fentanyl, and; 3) isotonitazene and naloxone are unable to partition on physiologically relevant timescales. Surprisingly, naloxone’s MFPT is orders of magnitude larger than morphine’s despite their highly similar structures.

Based on the probability flux and an effective reaction volume (13), we also estimated the membrane permeability coefficient *P_m_* [cm s^−1^] to compare with other drug molecules. Fentanyl has an estimated log*P_m_* around −7.5 (−7.3), which is much lower than the experimental log*P_m_* of water (−4) but only one order of magnitude lower than the small, neutral drug-like compounds zacopride, sotalo, and tacrine (between −5 and −6) (13). In contrast, the estimated log*P_m_* values of morphine and isotonitazene, −9.7 (−12.6) and −19.2 (−16.1), respectively, are comparable to the experimental log*P_m_* of potassium ions (−14) (19, 20), while the estimated log*P_m_* of naloxone is −36.9, which is orders of magnitude lower than all the opioid agonists.

### Membrane permeability is enhanced by early deprotonation within the membrane.

**Figure 2:**
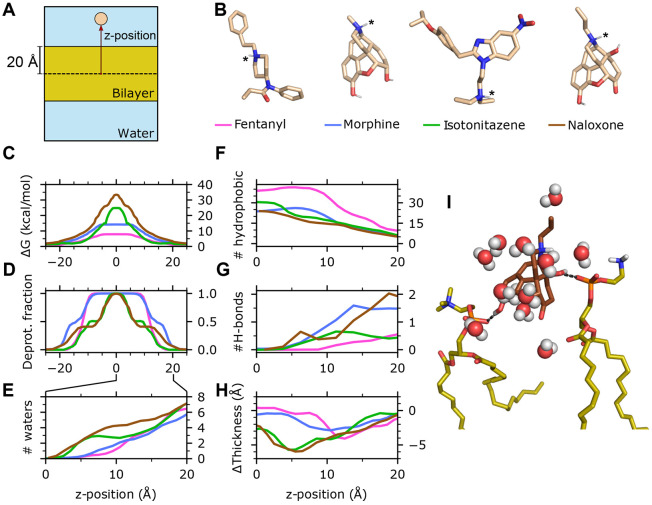
Molecular determinants of the distinct membrane permeability of fentanyl in comparison to morphine, isotonitazene, and naloxone. **A.** Illustration of the simulation system and *z* position of the permeant center of mass (COM). **B.** 3-D structures of the four compounds. Titratable nitrogen is indicated by an asterisk. Nonpolar hydrogens are hidden. The line color next to the permeant names corresponds to the data shown in **C-H**. **C.** Free energy profile (**C**) and deprotonation fraction (**D**) of the permeant along the permeant *z*-position. The profiles are symmetrized about *z* = 0 following Ref (13). **E.** Average number of water molecules within 3.4 Å from any heavy atom of the permeant as a function of *z*. **F,G.** Number of hydrophobic contacts (**F**) and H-bonds (**G**) between the permeant and lipid molecules as a function of *z*. **H.** Change of the membrane local thickness around the permeant as a function of *z*. The local thickness is defined as the *z* distance between the centers of phosphorous atoms in the upper and lower leaflets with a 10-Å cylinder around the permeant COM. The average value of the local thickness when the permeant COM is > 30 Å from the membrane is used as a reference. Data for **C-H** are taken from the final 100 iterations of the second set of WE-CpHMD simulations (see Figure S6 for the first set of simulations). **I.** A trajectory snapshot shows that naloxone (brown) forms two H-bonds between its hydroxyl groups and the phosphate groups of two lipids while its charged amine interacts with several water molecules.

To understand the distinct membrane permeation behavior of the four molecules, we examined several microscopic quantities of the permeant as a function of its *z*-position, including the free energy profile (i.e., potential of mean force), fraction of deprotonation, change in the local membrane thickness, number of the first-solvation-shell water molecules, number of hydrogen bonds (H-bonds) and hydrophobic contacts (Fig. 2). The *z*-dependent free energy (FE) profiles display a maximum at the membrane center for all molecules studied (Fig. 2C). Consistent with the trend in permeation rates, fentanyl exhibits the lowest FE barrier at 7.8 (11.2) kcal/mol, followed by morphine at 14.2 (18.8) kcal/mol, then isotonitazene at 24.8 (20.1) kcal/mol, and finally naloxone with the highest barrier at 33.4 (38.9) kcal/mol. To understand how these molecules that are charged in solution enter the hydrophobic membrane core, we examined the deprotonation profiles along *z* (Fig. 2D). Fentanyl remains protonated when its COM is close to the phosphate headgroups (*z* ≈ 20 Å) and begins deprotonation around 15 Å, becoming fully deprotonated below *z* ≈ 10 Å. Morphine begins deprotonation earlier, just below the phosphate headgroups, and becomes fully deprotonated slightly later than fentanyl, below *z* ≈ 7 Å. Despite sharing a highly similar structure with morphine, the titration profile of naloxone resembles that of isotonitazene, with early deprotonation onset but complete deprotonation only near the membrane center. These data demonstrate that the ability of the weakly basic permeant to complete deprotonation in the membrane is correlated with its permeability.

### Hydrophobicity and diminished hydrogen bonding capacity drive the distinct titrationdependent permeation behavior of fentanyl.

In solution, the charged amine of the four molecules is stabilized through hydrogen bonding (H-bonding) with water. During titration-coupled membrane partitioning, the number of water molecules surrounding the permeant decreases. Fentanyl and morphine become completely desolvated below *z* ≈ 5 Å, while isotonitazene and naloxone retain 1–2 water molecules until reaching closer to the bilayer center (Fig. 2E and SI Fig. S6). The earlier desolvation of fentanyl and morphine within the membrane is consistent with their earlier deprotonation compared to isotonitazene and naloxone. This raises the question, why does fentanyl permeate the membrane drastically faster compared to all other molecules? Important clues are provided by the hydrophobic and H-bond interaction profiles along *3* (Fig. 2F and G; SI Fig. S6), which correlate with the titration profiles. Throughout the membrane partitioning process, fentanyl forms the largest number of hydrophobic contacts in comparison to all other molecules, starting at about 10 and increasing to about 40 below *z* ≈ 10 Å, compared to 5–25 for morphine. This corroborates its 700-fold higher P_ow_ relative to morphine (8). At the same time, fentanyl forms the least number of H-bonds with lipids, with an average occupancy of 0.5 at the level of the phosphate group and decreases to zero below *z* ≈10 Å. In contrast, the hydroxyl groups of morphine and naloxone are capable of forming H-bonds with the phosphate groups of two lipids at the membrane-water interface, and one H-bond persists until *z* approaches 5 Å (Fig. 2I).

### Interactions between the charged amine and water slows down permeation and causes local membrane distortion.

Given the nearly identical structures and the similar number hydrophobic and H-bonding contacts, the significantly slower permeation rate of naloxone relative to morphine is puzzling. Simulations revealed that the delayed titration of naloxone is correlated with the persistent interactions between the charged amine and water molecules until approaching the bilayer center (Fig. 2D). Since these water molecules simultaneously interact with surrounding lipids (Fig. 2I), local lipids are displaced downward, reducing local membrane thickness. Interestingly, upon naloxone deprotonation, the membrane thickness recovers to normal values (Fig. 2H). The relationship between the hydration of the charged amine of naloxone and membrane thinning is corroborated by the first set of WE-CpHMD simulations, where the membrane exhibits progressive thinning as naloxone moves toward the bilayer center while preserving interactions between its charged amine and two water molecules (SI Fig. S6). The delayed titration of isotonitazene is also correlated with the persistent interactions with water and the resulting local membrane thinning (Fig. 2D,E,H). We suggest that the persistent interaction between the charged amine and water molecules is another significant contributor to the reduced permeation rates of naloxone and isotonitazene.

### Fentanyl inserts vertically at the membrane-water interface but adopts random orientations within the bilayer.

**Figure 3:**
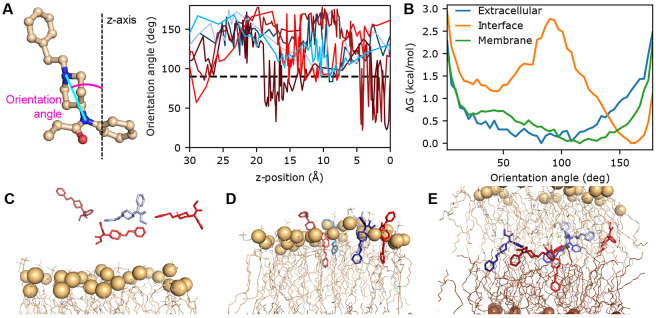
Fentanyl inserts vertically into the bilayer and adopts various orientations in the membrane. **A.** The orientational angle of fentanyl (left) as a function of *z* is calculated for unique continuous trajectories of fentanyl (right) permeating through the lipid bilayer. Angles greater than 90° show the phenethyl group oriented downward. **B.** The free energy profile along the orientation angle while fentanyl is in three different regions: extracellular (blue; defined by when the z-position is between 31-35 Å), at the upper leaflet interface (orange; when the z-position is between 18-22 Å), and in the middle of the membrane (green; when the z-position is between −2-2 Å). **C-E.** Snapshots selected from the seven trajectories, showing insertion into the upper leaflet (**C**, phosphorous atoms in tan), passage through the center of the bilayer (**D**), and insertion into the lower leaflet (**E**, phosphorous atoms in brown). Fentanyl is colored based on the trajectory the snapshot originated, matching **A**.

To further understand the extraordinary membrane permeability of fentanyl, we analyzed its conformational dynamics during the permeation process. The orientation of fentanyl is defined using an angle formed between the membrane normal and a vector drawn from the amide nitrogen to the piperidine amine nitrogen (Fig. 3A). In solution, fentanyl samples random orientations (Fig. 3A-C and SI Fig. S7); as it initiates the partitioning process at the bilayer-water interface (*z* ~20 Å), a vertical orientation relative to the membrane (angle around 30° or 160°) is preferred, as demonstrated by the trajectory snapshots and the bimodal free energy profiles (Fig. 3A,B,D and SI Fig. S7). This vertical orientational preference may reflect the elongated molecular geometry of fentanyl, which exhibits greater compatibility with lipid organization than the globular ‘T-shaped’ structure of morphine or naloxone, thereby facilitating rapid membrane permeation. Note, while both sets of WE-CpHMD simulations favor vertical orientations for fentanyl, the second set shows a modest preference for the downward conformation, where the phenethyl group is oriented toward the membrane interface (Fig. 3A,B,D). Interestingly, once past the lipid head groups, fentanyl is free to adopt other orientations, and in the middle of the bilayer, no orientational preference is observed (Fig. 3B,E and SI Fig. S7).

### Fentanyl is retained by the cell membrane and can reactivate the receptor even after washout and in competition with naloxone.

**Figure 4:**
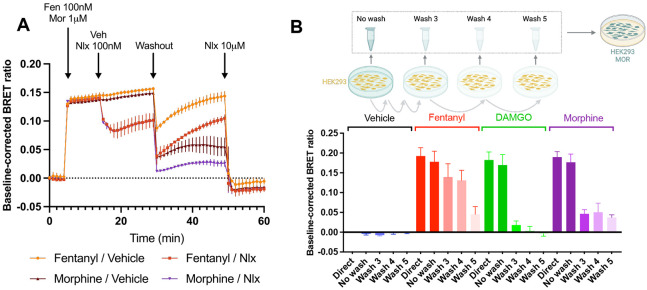
Experimental data shows fentanyl is retained by the cell membrane. **A.** Cells were first transfected with *μ*OR and Gi G protein activation sensor. After reading the baseline, cells were stimulated with either 100 nM fentanyl or 1 *μ*M morphine. 10 minutes later 100 nM naloxone or vehicle was introduced, followed by a three consecutive washouts. The cells were monitored for 20 minutes before finally introducing a high dose of 10 *μ*M naloxone. **B.** Untransfected cells were incubated with a 10 *μ*M concentration of vehicle (black), fentanyl (red), DAMGO (green), or morphine (purple); the supernatant of the cells was recovered either immediately (No Wash) or after a series of 3 to 5 washes (Wash 3-5). The supernatant was then introduced to simulate separate cells transfected with the *μ*OR and G_i_ G protein activation sensor as in **A**. For comparison, the stimulation from a direct application of the 10 *μ*M vehicle or opioid (Direct) was also measured.

To experimentally verify the unique membrane permeability of fentanyl, we developed a sensitive bioluminescence resonance energy transfer (BRET) protocol that harnesses the ability of MOR to efficiently activate G_i_ G proteins upon binding of an opioid agonist. Cells expressing this BRET sensor were first stimulated with either fentanyl or morphine, then either 100 nM naloxone or the vehicle was injected. After some time, three washouts were performed consecutively; these washouts were intended to completely remove any opioid agonist or naloxone even if it is initially bound to the receptor. The cells were then monitored for an additional 20 minutes, after which a high concentration of 10 *μ*M naloxone was applied. Fig. 4A shows the G-protein activation following the events described above. Following the three washouts, fentanyl shows substantial reactivation of the G protein complex–regardless if naloxone (red line) or an empty vehicle (yellow) was previously applied. Morphine, in contrast, shows minimal reactivation (brown and purple lines). These observations suggest that fentanyl is retained by the cell membrane to a greater degree than morphine and is capable of reactivating the *μ*OR.

To further test if cells can retain fentanyl, a separate experiment was conducted where non-transfected cells were exposed to a 10 *μ*M concentration of either fentanyl or morphine for 30 minutes. In these experiments the stable opioid peptide DAMGO was used as a control, known to be impermeable to the cell membrane. The supernatant was then extracted, either immediately (No Wash) or after a series of 3-5 washouts (Wash 3, Wash 4, and Wash 5). Reporter cells described above were then exposed to this supernatant, and the measured activation was compared to a direct exposure to a 10 *μ*M concentration of the corresponding opioid (Direct, Fig. 4B). Not surprisingly, the activation of No Wash and Direct were similar for all opioid agonists; after 3 washes, however, the response for morphine- and DAMGO-incubated cells is significantly lower than the effect of direct application, demonstrating the removal of agonist by the washouts. In contrast, after 3 washes of cells exposed to fentanyl, a response similar to directly applied fentanyl remains. In fact, for fentanyl a significant drop in activation is only seen in Wash 5. This suggests that fentanyl is more resilient to washouts than morphine and DAMGO, which we hypothesize is due to its ability to partition into the cell membrane.

### IAM chromatography shows fentanyl has a significantly higher affinity for phospholipids compared to morphine and naloxone.

Finally, we conducted Immobilized Affinity Membrane (IAM) chromatography to experimentally estimate the affinity of fentanyl for the phospholipid bilayer relative to morphine and naloxone. IAM chromatography phases are prepared from POPC analogs to closely mimic the hydrophobic, electrostatic, and H-bond interactions of drugs with biological membranes, which are collectively known as phospholipophilicity. Chromatographic Hydrophobicity Index (CHI), which measures the retention of the analyte in the column relative to the mobile phase, provides a more accurate estimate of drug-membrane affinity than the octanol-water partition coefficient (P_ow_). Our estimated CHI for fentanyl is 41, compared to 17 and 28 for morphine and naloxone, demonstrating that fentanyl has a significantly higher affinity for phospholipids (Table 1). However, the CHI value for naloxone is somewhat higher than morphine, which seemingly contradicts the significantly lower permeability of naloxone estimated from our simulations. This discrepancy may be explained by two key differences between the experiment and simulations. First, in the experiment, analytes can interact with phosphate headgroups and be retained in that region, thereby increasing retention time. In contrast, simulated permeability requires the molecule to reach the membrane’s hydrophobic core. In fact, naloxone forms slightly more h-bonds with the phosphate headgroups than morphine (Fig. 2G). Second, the IAM column is composed of a monolayer mimicking POPC lipids with C14 saturated chains and a phosphatidylcholine headgroup attached via a glycerol linker, whereas our simulated bilayer consists of a POPC/POPE/cholesterol mixture. A recent MD study found POPC membranes to be significantly more permeable to ethanol than POPE (21), suggesting that the experimental system may exhibit higher membrane affinity for both morphine and naloxone than what was found in our simulations.

## Discussion

Our simulations and experimental data suggest that fentanyl has an exceptional affinity for the cell membrane. The simulation-estimated effective *P_m_* of fentanyl at pH 7.5 is on the order of 10^−7^ cm/s, which is at least two orders of magnitude larger than morphine. Our estimated permeability of fentanyl is 1–2 orders of magnitude smaller than nicotine, estimated as 10^−6^ cm/s at pH 7.4 (22) and 10^−5^ cm/s at pH 7.8 (23) by two independent studies. This difference is justifiable, considering that nicotine has a much smaller size but titrates at physiological pH with a similar p*K*_a_ value of 7.9 (23). Note, compared with nicotine, similarly sized neutral drug-like compounds, zacopride, sotalo, and tacrine have similar permeability range of 10^−5^−10^−6^ cm/s (12). Consistent with the simulation results, our experiments showed that fentanyl can reactivate the *μ*OR following washout and after naloxone displacement, whereas morphine cannot. These findings support the hypothesis that the membrane acts as a drug reservoir for fentanyl, either elevating local fentanyl concentrations or facilitating receptor binding through an alternative lipid-mediated pathway. This membrane-dependent mechanism may significantly contribute to the extreme potency of fentanyl relative to morphine.

While most drugs are at least partially ionized in solution at physiological pH (24, 25), the long-standing pH partition hypothesis posits that only the neutral form can traverse biological membranes (26). This hypothesis is supported by pH-dependent permeability profiles observed for ionizable drugs. For example, nicotine (p*K*_a_ values of 7.9) (23) demonstrates increasing *P_m_* with increasing pH (22, 23), corresponding to a greater fraction of the neutral form. In support of the pH partition hypothesis, our simulations demonstrated that only neutral species can cross the membrane; however, our simulations additionally revealed a more nuanced mechanism: weakly basic drugs can initially partition into the lipid environment in the protonated form and subsequently undergo deprotonation prior to reaching the bilayer center. This proton-coupled permeation mechanism is kinetically feasible because the timescale of deprotonation events, which is experimentally estimated at 1-10 *μ*s based on *K*_off_ on the order 1–10 x 10^5^ s^−1^ (27), is orders of magnitude faster than that of membrane permeation events of most permeable drugs, which is estimated as 0.4 – 40 ms based on the effective permeability range of 10^−7^ – 10^−5^ cm/s at physiological pH (15, 28) and a membrane thickness of 40 Å.

To contextualize our findings, we compare our simulation results with previous theoretical studies of membrane permeation of small ionizable drugs using umbrella sampling PMF calculations based on either fixed-charge simulations (17, 29) or membrane-enabled hybrid-solvent CpHMD (16). Notably, fixed-charge simulations assume that ionized drugs can permeate the membrane, albeit at reduced rates, and thus the overall effective permeability at specific pH is determined by the fraction of the neutral species in solution (16, 17, 29). Our results align with the PMF calculations of propranolol (p*K*_a_ of 9.5) partitioning into the membrane using hybrid-solvent CpHMD simulations (16) in demonstrating that ionizable drugs neutralize as they approach the hydrophobic membrane core. These observations are consistent with the experimental data (30) and multi-site *λ*-dynamics simulations (31) showing that ionizable residues in membrane-inserted peptides (with the exception of arginine) undergo large p*K*_a_ shifts that allow them to adopt the neutral state at physiological pH. Arginine maintains its positive charge, consequently inducing pore formation and membrane deformation (30, 32); consistent with this, our simulations demonstrated that naloxone and isotonitazene, which remain partially protonated until reaching the membrane core, induce localized hydration and membrane thinning. Note, an important contrast between our results and the hybrid-solvent CpHMD umbrella sampling simulations of propranolol (16) is that our calculated PMFs consistently exhibit energy barriers at the membrane center, which is in agreement with both fixed-charge umbrella sampling studies (17, 29) and the weighted ensemble permeation simulations of neutral molecules (13).

The present simulations have several caveats. While the relative permeability ranking among the four molecules is robust, the uncertainty in *P_m_* values for poorly permeable molecules is substantial due to insufficient sampling of barrier-crossing events which are extremely rare. Notably, in the first set of simulations for naloxone, no permeating event was observed. Additionally, although WE simulations are theoretically unbiased, the selection of progress variables and other protocol parameters can influence the estimated *P_m_* values, as demonstrated previously (13). Another limitation is the use of additive force field, which is known to overestimate the hydrophobicity of alkane environments such as the bilayer core. This would affect the precise membrane depth at which complete deprotonation occurs, although the qualitative trends should remain valid. There are caveats with the performed experiments. Whilst IAM chromatography is more representative of the phospholipid membrane compared to P_ow_ estimates, there are still differences between the lipid composition and arrangement on the column compared to a cell membrane bilayer. Furthermore, the IAM chromatography approach is not able to incorporate other membrane constituents such proteins, cholesterol, glycolipids etc. Nevertheless, the present work offers compelling evidence to support the notation that plasma membrane accumulation is an important driver of fentanyl’s extreme potency, establishing a foundation for future studies to investigate the membrane-dependent mechanism of action of fentanyl and other opioids. Understanding membrane-dependent pharmacology has implications for designing new opioid antagonists.

## Supplementary Material

Supplement 1

## Figures and Tables

**Table 1:** Effective permeability and mean-first passage time estimated from simulations and measured chromatographic hydrophobicity index. Estimated effective permeability (log*P_m_*) and mean first passage time (MFPT) from the WE-CpHMD simulations (top, second trial; bottom, first trial) as well as the measured chromatographic hydrophobicity index (CHI) from IAM experiments (isotonitazene not measured). The permeability and shown range were calculated using the average and standard error of the probability flux from extracellular to intracellular regions. For naloxone, log*P_m_* and MFPT could not be estimated in the first trial due to the absence of permeation events (SI Fig. S3).

	log(*P_m_*[cm s^−1^])	MFPT (s)	CHI
Fentanyl	−7.7 [−7.9,−7.5]	10. [7.4,16]	41
	−7.3 [−7.5,−7.2]	4.6 [3.4,6.9]	
Morphine	−9.6 [−9.9,−9.5]	9.8e2 [6.2e2,1.8e3]	17
	−12.6 [−12.7,−12.5]	8.8e5 [7.1e5,1.2e6]	
Isotonitazene	−19.2 [−19.7,−19.0]	3.9e12 [2.3e12, 1.1e13]	n/d
	−16.1 [−16.4,−15.9]	3.0e9 [1.9e9,6.2e9]	
Naloxone	−36.9 [−37.0,−36.8]	1.8e30 [1.5e30,2.4e30]	28

## References

[1] U.S. Overdose Deaths Decrease in 2023, First Time Since 2018, https://www.cdc.gov/nchs/pressroom/nchs_press_releases/2024/20240515.htm#print, accessed Oct 31, 2024.

[2] Zhuang Y., , Molecular Recognition of Morphine and Fentanyl by the Human *μ*-Opioid Receptor. Cell 185 (23), 4361–4375.e19 (2022), doi:10.1016/j.cell.2022.09.041. 36368306

[3] Che T., Roth B. L., Molecular Basis of Opioid Receptor Signaling. Cell 186 (24), 5203–5219 (2023), doi:10.1016/j.cell.2023.10.029. 37995655 PMC10710086

[4] Tsai M.-H. M., , In Vitro Functional Profiling of Fentanyl and Nitazene Analogs at the *μ*-Opioid Receptor Reveals High Efficacy for Gi Protein Signaling. ACS Chem. Neurosci. 15 (4), 854–867 (2024), doi:10.1021/acschemneuro.3c00750. 38345920 PMC11890208

[5] Comer S. D., Cahill C. M., Fentanyl: Receptor Pharmacology, Abuse Potential, and Implications for Treatment. Neurosci. Biobehav. Rev. 106, 49–57 (2019), doi:10.1016/j.neubiorev.2018.12.005. 30528374 PMC7233332

[6] Sutcliffe K. J., , Interaction With the Lipid Membrane Influences Fentanyl Pharmacology. Adv. Drug Alcohol Res. 2, 10280 (2022), doi:10.3389/adar.2022.10280. 35909438 PMC7613138

[7] Szlenk C. T., Gc J. B., Natesan S., Does the Lipid Bilayer Orchestrate Access and Binding of Ligands to Transmembrane Orthosteric/Allosteric Sites of G Protein-Coupled Receptors? Mol. Pharmacol. 96 (5), 527–541 (2019), doi:10.1124/mol.118.115113. 30967440 PMC6776015

[8] Roy S. D., Flynn G. L., Solubility and Related Physicochemical Properties of Narcotic Analgesics. Pharm. Res. 5 (9), 580–586 (1988), doi:10.1023/A:1015994030251. 2907788

[9] Huang Y., Chen W., Wallace J. A., Shen J., All-Atom Continuous Constant pH Molecular Dynamics With Particle Mesh Ewald and Titratable Water. J. Chem. Theory Comput. 12 (11), 5411–5421 (2016), doi:10.1021/acs.jctc.6b00552. 27709966 PMC5713900

[10] Harris J. A., , GPU-Accelerated All-Atom Particle-Mesh Ewald Continuous Constant pH Molecular Dynamics in Amber. J. Chem. Theory Comput. 18 (12), 7510–7527 (2022), doi:10.1021/acs.jctc.2c00586. 36377980 PMC10130738

[11] Huber G., Kim S., Weighted-Ensemble Brownian Dynamics Simulations for Protein Association Reactions. Biophys. J. 70 (1), 97–110 (1996), doi:10.1016/S0006-3495(96)79552-8. 8770190 PMC1224912

[12] Russo J. D., , WESTPA 2.0: High-Performance Upgrades for Weighted Ensemble Simulations and Analysis of Longer-Timescale Applications. J. Chem. Theory Comput. 18 (2), 638–649 (2022), doi:10.1021/acs.jctc.1c01154. 35043623 PMC8825686

[13] Zhang S., , Mechanistic Insights into Passive Membrane Permeability of Drug-like Molecules from a Weighted Ensemble of Trajectories. J. Chem. Inf. Model. 62 (8), 1891–1904 (2022), doi:10.1021/acs.jcim.1c01540. 35421313 PMC9044451

[14] Sun R., Dama J. F., Tan J. S., Rose J. P., Voth G. A., Transition-Tempered Metadynamics Is a Promising Tool for Studying the Permeation of Drug-like Molecules through Membranes. J. Chem. Theory Comput. 12 (10), 5157–5169 (2016), doi:10.1021/acs.jctc.6b00206. 27598403

[15] Bennion B. J., , Predicting a Drug’s Membrane Permeability: A Computational Model Validated With *in Vitro* Permeability Assay Data. J. Phys. Chem. B 121 (20), 5228–5237 (2017), doi:10.1021/acs.jpcb.7b02914. 28453293

[16] Yue Z., Li C., Voth G. A., Swanson J. M. J., Dynamic Protonation Dramatically Affects the Membrane Permeability of Drug-like Molecules. J. Am. Chem. Soc. 141 (34), 13421–13433 (2019), doi:10.1021/jacs.9b04387. 31382734 PMC6755907

[17] Harris J., Chipot C., Roux B., How Is Membrane Permeation of Small Ionizable Molecules Affected by Protonation Kinetics? J. Phys. Chem. B 128 (3), 795–811 (2024), doi:10.1021/acs.jpcb.3c06765. 38227958 PMC11702507

[18] Case D.A., , AMBER 2024 (2024).

[19] Yang N. J., Hinner M. J., Getting Across the Cell Membrane: An Overview for Small Molecules, Peptides, and Proteins. Methods Mol Biol 1266, 29–53 (2015), doi:10.1007/978-1-4939-2272-7_3. 25560066 PMC4891184

[20] Papahadjopoulos D., Nir S., Ohki S., Permeability Properties of Phospholipid Membranes: Effect of Cholesterol and Temperature. Biochimica et Biophysica Acta (BBA) - Biomembranes 266 (3), 561–583 (1972), doi:10.1016/0005-2736(72)90354-9. 4625141

[21] Ghorbani M., Wang E., Krämer A., Klauda J. B., Molecular Dynamics Simulations of Ethanol Permeation through Single and Double-Lipid Bilayers. J. Chem. Phys. 153 (12), 125101 (2020), doi:10.1063/5.0013430. 33003717 PMC7656323

[22] Chen L.-L. H., Chetty D. J., Chien Y. W., A Mechanistic Analysis to Characterize Oramucosal Permeation Properties. Int. J. Pharm. 184 (1), 63–72 (1999), doi:10.1016/S0378-5173(99)00091-5. 10425352

[23] Nielsen H. M., Rassing M. R., Nicotine Permeability across the Buccal TR146 Cell Culture Model and Porcine Buccal Mucosa in Vitro: Effect of pH and Concentration. Eur.J.Pharm.Sci. 16 (3), 151–157 (2002), doi:10.1016/s0928-0987(02)00083-0. 12128169

[24] Manallack D. T., Prankerd R. J., Yuriev E., Oprea T. I., Chalmers D. K., The Significance of Acid/Base Properties in Drug Discovery. Chem. Soc. Rev. 42 (2), 485–496 (2013), doi: 10.1039/C2CS35348B. 23099561 PMC3641858

[25] Charifson P. S., Walters W. P., Acidic and Basic Drugs in Medicinal Chemistry: A Perspective. J. Med. Chem. 57 (23), 9701–9717 (2014), doi:10.1021/jm501000a. 25180901

[26] Hollinger M. A., Introduction to Pharmacology (CRC Press, Boca Raton), 3 ed. (2007), doi: 10.1201/b13963.

[27] Wallerstein J., Weininger U., Khan M. A. I., Linse S., Akke M., Site-Specific Protonation Kinetics of Acidic Side Chains in Proteins Determined by pH-Dependent Carboxyl ^13^ C NMR Relaxation. J. Am. Chem. Soc. 137 (8), 3093–3101 (2015), doi:10.1021/ja513205s. 25665463

[28] Pade V., Stavchansky S., Link between Drug Absorption Solubility and Permeability Measurements in Caco-2 Cells. J. Pharm. Sci. 87 (12), 1604–1607 (1998), doi:10.1021/js980111k. 10189274

[29] Oung S. W., Kremer N., Ben Amara S., Zaidi A., Koslowski T., Protonation and Orientation: A Computational Approach to Cocaine Diffusion through a Model Membrane. Phys. Chem. Chem. Phys. 24 (23), 14219–14227 (2022), doi:10.1039/D2CP01140A. 35647789

[30] Gleason N. J., Vostrikov V. V., Greathouse D. V., Koeppe R. E., Buried Lysine, but Not Arginine, Titrates and Alters Transmembrane Helix Tilt. Proc. Natl. Acad. Sci. USA 110 (5), 1692–1695 (2013), doi:10.1073/pnas.1215400110. 23319623 PMC3562795

[31] Panahi A., Brooks C. L., Membrane Environment Modulates the p *K* _a_ Values of Transmembrane Helices. J. Phys. Chem. B 119 (13), 4601–4607 (2015), doi:10.1021/acs.jpcb.5b00289. 25734901 PMC4404502

[32] Herce H., , Arginine-Rich Peptides Destabilize the Plasma Membrane, Consistent with a Pore Formation Translocation Mechanism of Cell-Penetrating Peptides. Biophys. J. 97 (7), 1917–1925 (2009), doi:10.1016/j.bpj.2009.05.066. 19804722 PMC2756373

[33] Thurlkill R. L., Cross D. A., Scholtz J. M., Pace C. N., pKa of Fentanyl Varies With Temperature: Implications for Acid-Base Management During Extremes of Body Temperature. J. Cardiothorac. Vasc. Anesth. 19 (6), 759–762 (2005), doi:10.1053/j.jvca.2004.11.039. 16326301

[34] Budavari S., O’Neil. M., Smith A., Henckelman P., Obenchain J., The Merck Index: 12th Edition 1996 (CRC Press), 12 ed. (1996).

[35] Oberst F. W., Andrews H. L., The Electrolytic Dissociation of Morphine Derivatives and Certain Synthetic Analgetic Compounds. J. Pharmacol. Exp. Therap. 71 (1), 38–41 (1941), doi:10.1016/S0022-3565(25)10069-4.

[36] Kaufman J. J., Semo N. M., Koski W. S., Microelectrometric Titration Measurement of the pKa’s and Partition and Drug Distribution Coefficients of Narcotics and Narcotic Antagonists and Their pH and Temperature Dependence. J. Med. Chem. 18 (7), 647–655 (1975), doi: 10.1021/jm00241a001. 239235

[37] Henderson J. A., , A Guide to the Continuous Constant pH Molecular Dynamics Methods in Amber and CHARMM [Article v1.0]. Liv. J. Comput. Mol. Sci. 4 (1), 1563 (2022), doi: 10.33011/livecoms.4.1.1563. PMC9910290 36776714

[38] Vanommeslaeghe K., MacKerell A. D., Automation of the CHARMM General Force Field (CGenFF) I: Bond Perception and Atom Typing. J. Chem. Inf. Model. 52 (12), 3144–3154 (2012), doi:10.1021/ci300363c. 23146088 PMC3528824

[39] Vanommeslaeghe K., Raman E. P., MacKerell A. D., Automation of the CHARMM General Force Field (CGenFF) II: Assignment of Bonded Parameters and Partial Atomic Charges. J. Chem. Inf. Model. 52 (12), 3155–3168 (2012), doi:10.1021/ci3003649. 23145473 PMC3528813

[40] Jorgensen W. L., Chandrasekhar J., Madura J. D., Comparison of Simple Potential Functions for Simulating Liquid Water. J. Chem. Phys. 79 (2), 926 (1983), doi:10.1063/1.445869.

[41] MacKerell A. D. Jr., , All-Atom Empirical Potential for Molecular Modeling and Dynamics Studies of Proteins. J. Phys. Chem. B 102 (18), 3586–3616 (1998), doi:10.1021/jp973084f. 24889800

[42] Beglov D., Roux B., Finite Representation of an Infinite Bulk System: Solvent Boundary Potential for Computer Simulations. J. Chem. Phys. 100 (12), 9050–9063 (1994), doi:10.1063/1.466711.

[43] Lev B., Roux B., Noskov S. Y., Relative Free Energies for Hydration of Monovalent Ions from QM and QM/MM Simulations. J. Chem. Theory Comput. 9 (9), 4165–4175 (2013), doi:10.1021/ct400296w. 26592407

[44] Essmann U., , A Smooth Particle Mesh Ewald Method. J. Chem. Phys. 103 (19), 8577–8593 (1995), doi:10.1063/1.470117.

[45] Åqvist J., Wennerström P., Nervall M., Bjelic S., Brandsdal B. O., Molecular Dynamics Simulations of Water and Biomolecules with a Monte Carlo Constant Pressure Algorithm. Chem. Phys. Lett. 384 (4-6), 288–294 (2004), doi:10.1016/j.cplett.2003.12.039.

[46] Jo S., Kim T., Iyer V. G., Im W., CHARMM-GUI: A Web-Based Graphical User Interface for CHARMM. J. Comput. Chem. 29 (11), 1859–1865 (2008), doi:10.1002/jcc.20945. 18351591

[47] Calderon R. O., Attema B., DeVries G. H., Lipid Composition of Neuronal Cell Bodies and Neurites from Cultured Dorsal Root Ganglia. J. Neurochem. 64 (1), 424–429 (1995), doi: 10.1046/j.1471-4159.1995.64010424.x. 7798942

[48] Klauda J. B., , Update of the CHARMM All-Atom Additive Force Field for Lipids: Validation on Six Lipid Types. J. Phys. Chem. B 114, 7830–7843 (2010). 20496934 10.1021/jp101759q PMC2922408

[49] McGibbon R. T., , MDTraj: A Modern Open Library for the Analysis of Molecular Dynamics Trajectories. Biophys. J. 109 (8), 1528–1532 (2015), doi:10.1016/j.bpj.2015.08.015. 26488642 PMC4623899

[50] Bogetti A. T., , A Suite of Advanced Tutorials for the WESTPA 2.0 Rare-Events Sampling Software [Article v2.0]. LiveCoMS 5 (1) (2022), doi:10.33011/livecoms.5.1.1655. PMC10191340 37200895

[51] Valko K., Du C. M., Bevan C. D., Reynolds D. P., Abraham M. H., Rapid-Gradient HPLC Method for Measuring Drug Interactions with Immobilized Artificial Membrane: Comparison with Other Lipophilicity Measures. J. Pharm. Sci. 89 (8), 1085–1096 (2000), doi:10.1002/1520-6017(200008)89:8⟨1085::AID-JPS13⟩3.0.CO;2-N. 10906732

[52] Hollósy F., , Estimation of Volume of Distribution in Humans from High Throughput HPLC-Based Measurements of Human Serum Albumin Binding and Immobilized Artificial Membrane Partitioning. J. Med. Chem. 49 (24), 6958–6971 (2006), doi:10.1021/jm050957i. 17125249

[53] Wang R., Fu Y., Lai L., A New Atom-Additive Method for Calculating Partition Coefficients. J. Chem. Inf. Comput. Sci. 37 (3), 615–621 (1997), doi:10.1021/ci960169p.

[54] Alexander S. P. H., , The Concise Guide to PHARMACOLOGY 2023/24: G Protein-Coupled Receptors. Br. J. Pharmacol. 180 (S2), S23–S144 (2023), doi:10.1111/bph.16177. 38123151 PMC13324819

[55] Willighagen E. L., , The Chemistry Development Kit (CDK) v2.0: Atom Typing, Depiction, Molecular Formulas, and Substructure Searching. J. Cheminform. 9 (1), 33 (2017), doi:10.1186/s13321-017-0220-4. 29086040 PMC5461230

