# Supplementary material for: Membrane Permeability Drives the Extreme Potency of Fentanyl but not Isotonitazene
Source: bioRxiv. 2025 Oct 16:2025.07.22.666146. Originally published 2025 Jul 26. Preprint. [Version 3] doi: 10.1101/2025.07.22.666146 (PMC12330492; doi:10.1101/2025.07.22.666146)
Supplement: Supplement 1 [file media-1.pdf]

Supporting Information: Membrane Permeability Drives the Extreme Potency of Fentanyl but not Isotonitazene

Joseph Clayton,[†,‡] George J. Farmer,[¶,§,‖] Jacqueline Glenn,[§,‖] Shailesh N. Mistry,[¶,§,‖] J. Robert Lane,[§,‖] Lei Shi,[⊥] Lidiya Stavitskaya,[†] Meritxell Canals,[§,‖] and Jana Shen[*,‡]

† *Division of Applied Regulatory Science, Office of Clinical Pharmacology, Office of Translational Sciences, Center for Drug Evaluation and Research, United States Food and Drug Administration, Silver Spring, MD 20993, U.S.A.*

‡ *Department of Pharmaceutical Sciences, University of Maryland School of Pharmacy, Baltimore, MD 21201, U.S.A.*

¶ *Division of Biomolecular Science and Medicinal Chemistry, School of Pharmacy, and University of Nottingham Biodiscovery Institute, University Park, Nottingham, U.K.*

§ *Centre of Membrane Proteins and Receptors, Universities of Nottingham and Birmingham, Midlands, U.K.*

‖ *Division of Physiology, Pharmacology and Neuroscience, School of Life Sciences, University of Nottingham, Queens Medical Centre, Nottingham, U.K.*

⊥ *Computational Chemistry and Molecular Biophysics Section, National Institutes on Drug Abuse - Intramural Research Program, Baltimore, MD 21224, U.S.A.*

E-mail: jana.shen@rx.umaryland.edu

Supplemental Tables

Table S1: Parameters in the model titration PMF for the four compounds

Molecule	A	B
Fentanyl	-58.059565	0.100857
Morphine	-37.405443	0.136572
Isotonitazene	-51.327454	0.325572
Naloxone	-74.288134	0.046556

Table S2: Number of iterations and cumulative sampling time for each set of WE-CpHMD simulations

Molecule	First trial		Second trial	
	Iteration	Time	Iteration	Time (μs)
Fentanyl	550	14.9	500	15.7
Morphine	550	11.3	500	15.5
Isotonitazene	400	8.5	350	9.5
Naloxone	300	3.4	350	7.0

Table S3: Physicochemical properties of four studied compounds

Molecule	Mol weight	Rot. bonds	H-bond donor	H-bond acceptor	xLogP
Fentanyl	336	6	0	3	3.94
Isotonitazene	410	9	0	4	3.62
Morphine	285	0	2	2	0.49
Naloxone	327	2	3	2	0.31

Molecular weight is unit of g/mol. xlogP (calculated octanol-water partition coefficient)[S1] values are taken from the Database IUPAC/BPS Guide to Pharmacology[S2] which reports the CDK calculations.[S3]

Table S4: Hydrophobicity index from immobilized affinity membrane (IAM) experiments

Name	Acid/base profile	$t_R \pm$ SD (mins,n=6)	Measured CHI (IAM)	Reference CHI (IAM)
Propanolol	basic	3.30 ± 0.04	47	42
Nicardipine	basic	3.31 ± 0.03	47	45
Indomethacin	acidic	2.13 ± 0.06	27	30
Warfarin	acidic	1.56 ± 0.06	18	20
Carbamazepine	neutral	2.14 ± 0.02	27	27
Colchicine	neutral	1.80 ± 0.07	22	23

Components of suitability Test Mixtures 1 and 2 and their corresponding acid/base profile. The average $t_R \pm$SD from n=6 repeats measured using method 1 was used to determine CHI(IAM) for each molecule using the calibration plot (Figure S10). The column was deemed suitable for analysis due to measured CHI(IAM) values being within ±5 of reference CHI(IAM) from the literature.

Supplemental Figures

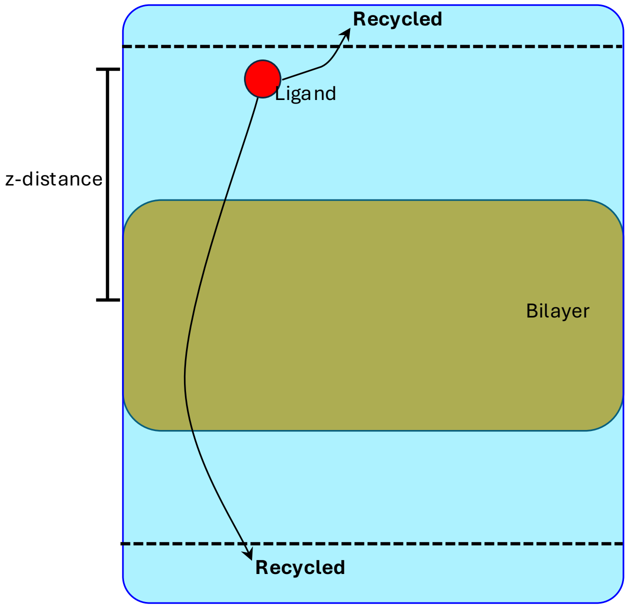

Figure S1: **Schematic of the steady-state condition.** Schematic of the steady-state condition used for the WE-CpHMD simulation. Once the ligand either moves far away from the membrane or permeates through, the walker is recycled–meaning the walker is terminated and a new one is started from the initial state.

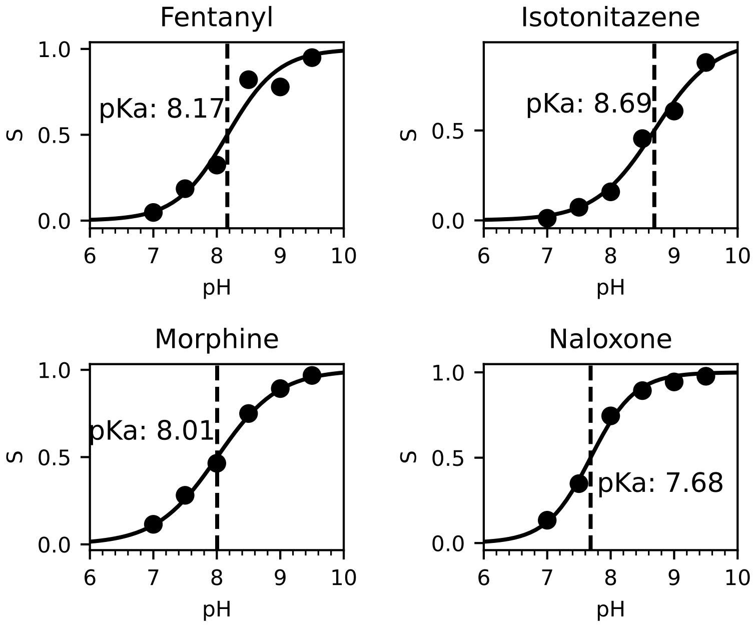

Figure S2: **Simulated solution titration curves of the four studied compounds.** Deprotonation fraction at different simulation pH conditions. 20 ns simulations were conducted at six different pH conditions ranging from 7.0 to 9.5. The final 10 ns of each was then used to calculate the deprotonation fraction. The calculated pK_a's are given.

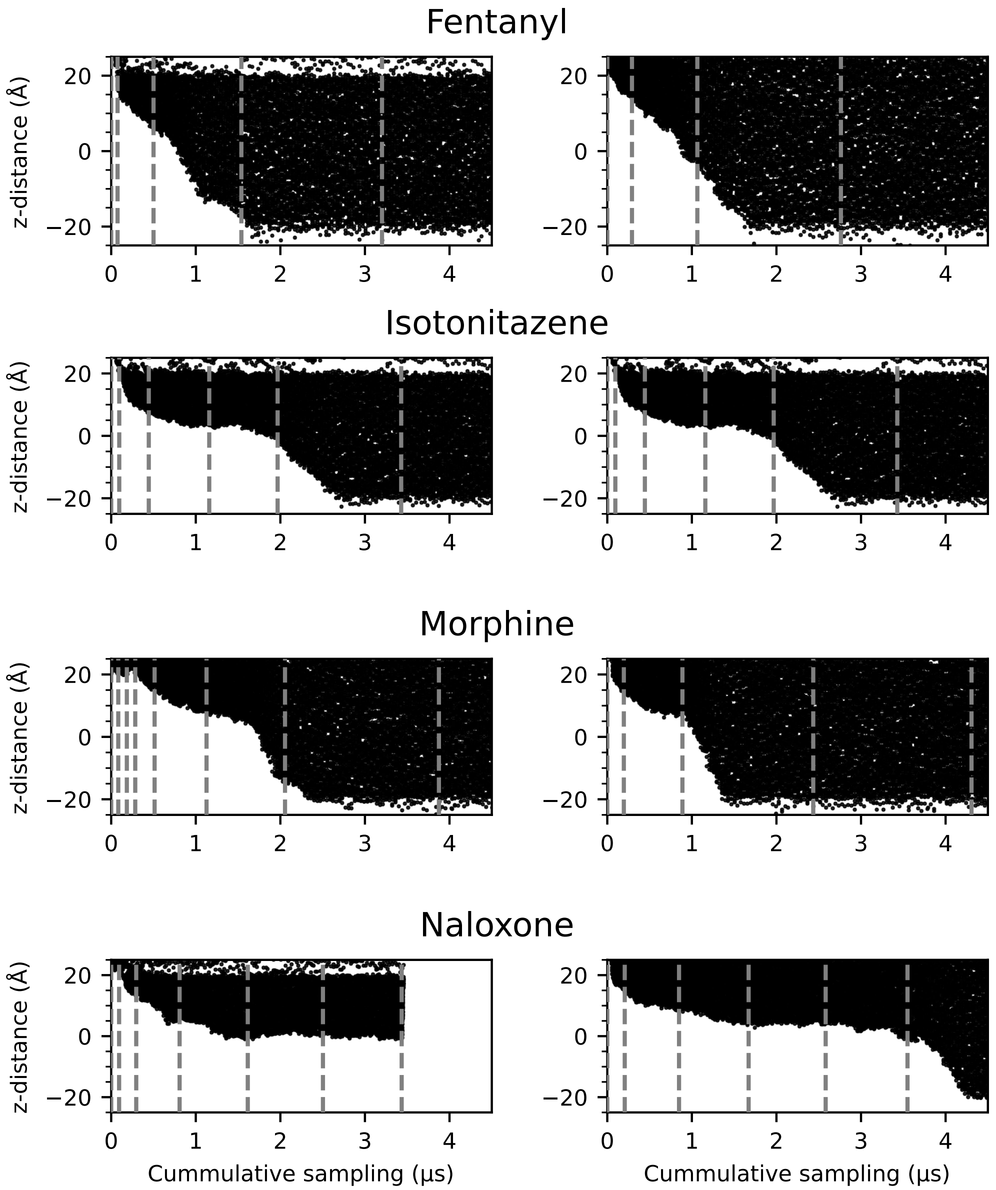

Figure S3: **Fentanyl requires the least simulation time to permeate the membrane.** z-position vs. cumulative simulation time of fentanyl, morphine, isotonitazene, and naloxone from the first (left) and second (right) sets of WE-CpHMD simulations. Gray dashed lines are drawn at every 50 WE iterations; for simplicity, only the first 4.5μs is shown.

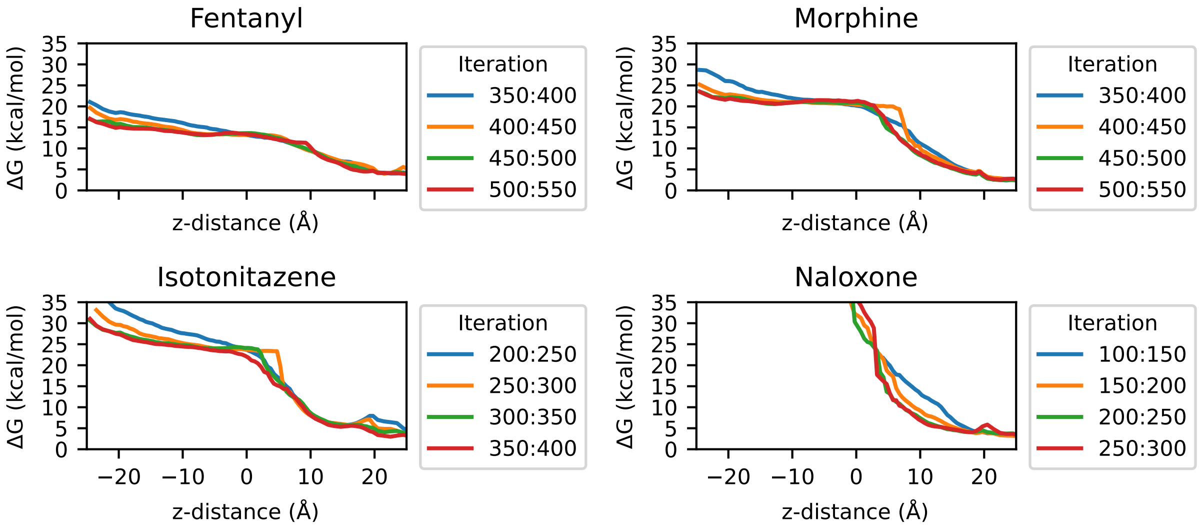

Figure S4: **Convergence of the potential of mean force calculations (first trial).** Convergence of the potential mean force, calculated using four windows containing 50 iterations, for the first set of weighted ensemble simulations.

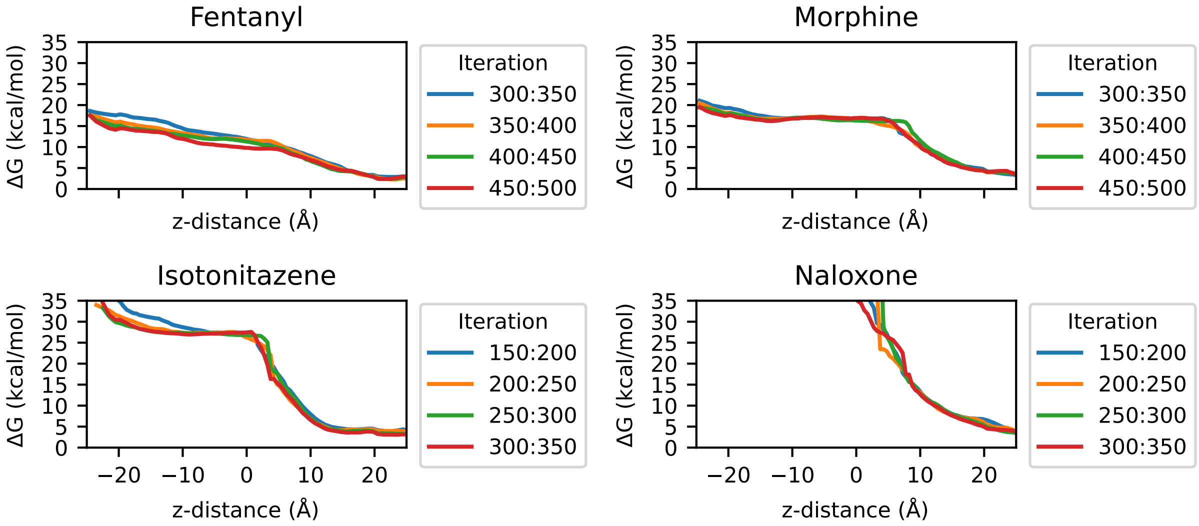

Figure S5: **Convergence of the potential of mean force calculations (second trial).** Convergence of the potential mean force, calculated using four windows containing 50 iterations, for the first set of weighted ensemble simulations.

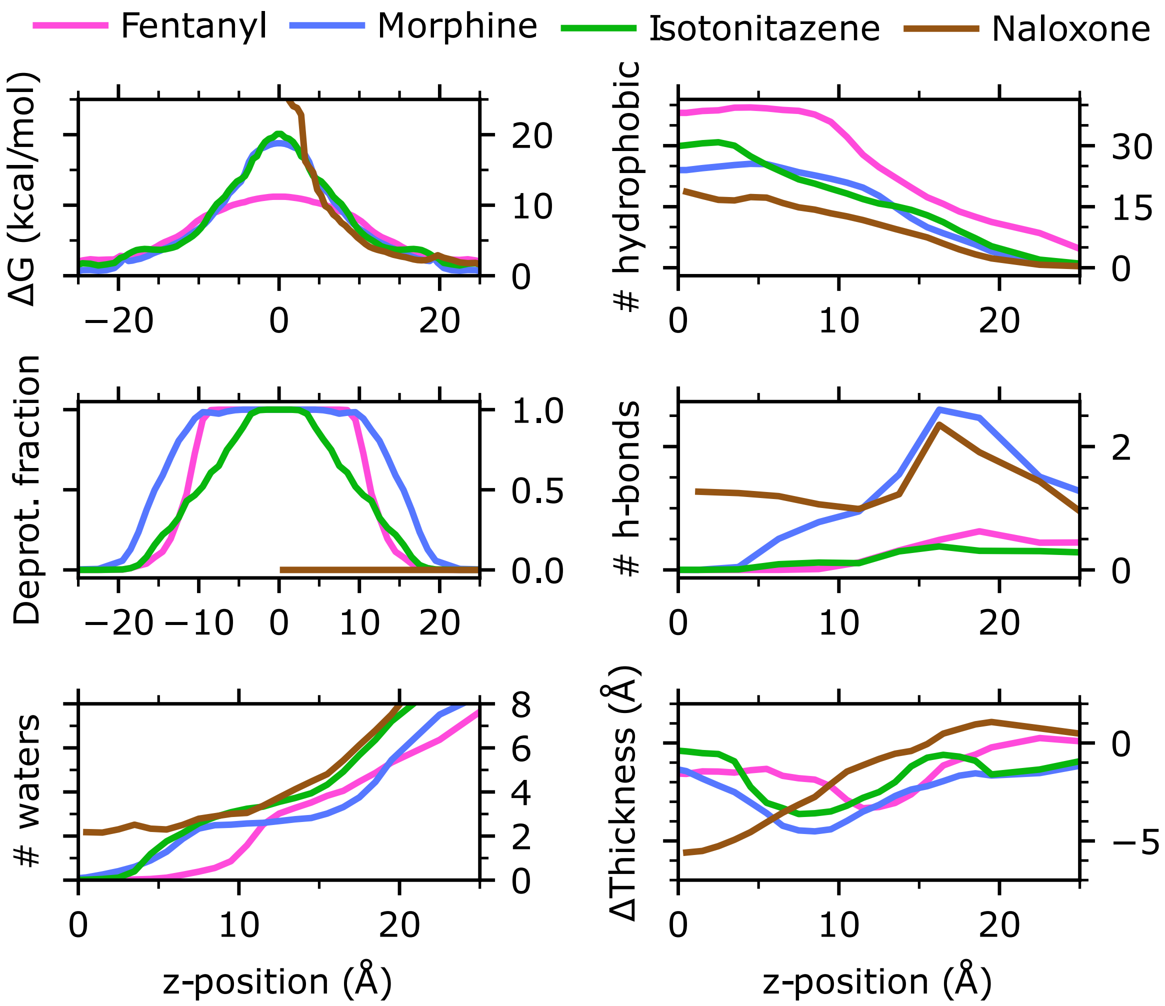

Figure S6: **Trends in permeation events are reproducible.** Characterization of the membrane permeation properties of fentanyl, morphine, isotonitazene, and naloxone from the simulation set 1. **Left.** Free energy profile (top), deprotonation fraction (middle) and the number of water within 3.4 Å from any heavy atom of the permeant (bottom) as a function of its z-position. The profiles are symmetrized about $z = 0$ following Ref.[S4] **Right.** Number of hydrophobic contacts (top) and h-bonds (middle) between the permeant and lipid molecules and the change of the membrane local thickness around the permeant (bottom) as a function of z. cylinder around the permeant COM. The average value of the local thickness when the permeant COM is > 30 Å from the membrane is used as a reference. Data are taken from the final 100 iterations of the first set of WE-CpHMD simulations.

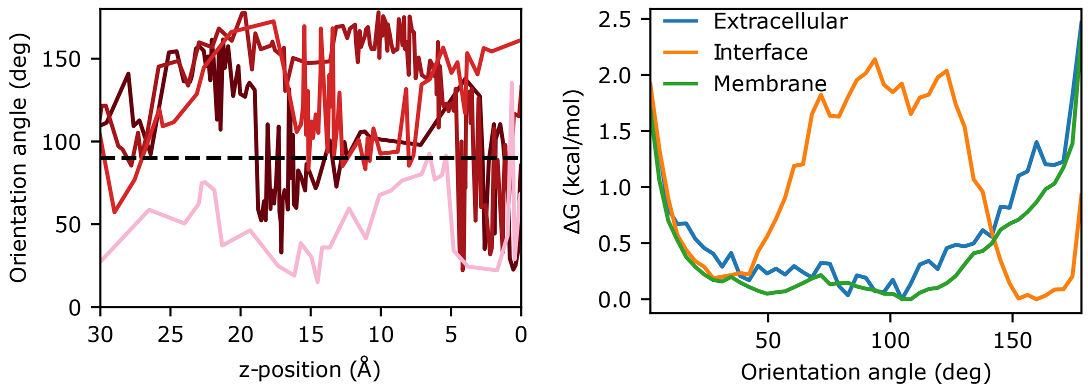

Figure S7: **Fentanyl prefers a vertical orientation at the interface in simulation set 1. Left.** Fentanyl orientation angle along stitched histories of walkers reaching the center of the membrane. **Right.** Potential mean force along the orientation angle while fentanyl is in the extracellular region, at the membrane interface, and within the membrane.

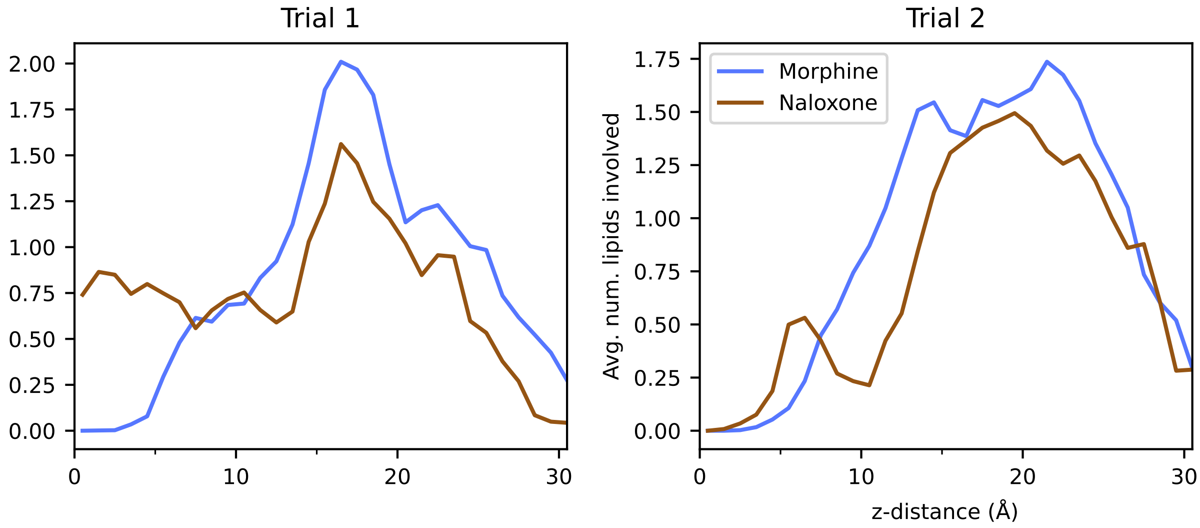

Figure S8: **Morphine and naloxone can interact with two separate phospholipid molecules while at the membrane interface.** Average number of lipids involved in hydrogen bonds with either morphine or naloxone, as a function of z-distance.

A

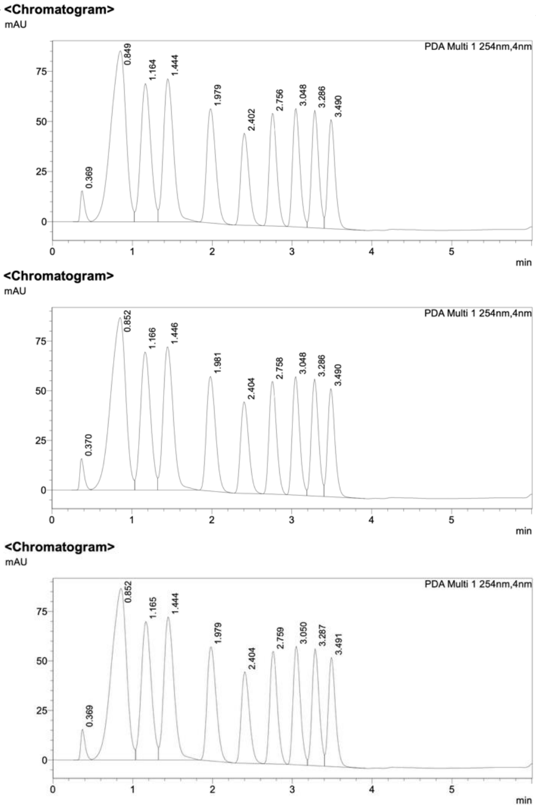

B

Calibration Standards	Structure	CHI (IAM)[54]	$t_R \pm$ SD (mins) n=8
octanophenone	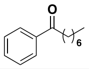	49.4	3.48 ± 0.04
heptanophenone	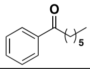	45.7	3.28 ± 0.04
hexanophenone	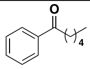	41.8	3.04 ± 0.04
valerophenone	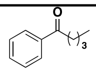	37.3	2.75 ± 0.04
butyrophenone	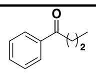	32.0	2.39 ± 0.04
propiophenone	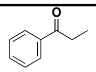	25.9	1.97 ± 0.04
acetophenone	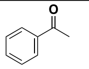	17.2	1.44 ± 0.04
acetanilide	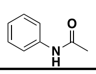	11.5	1.16 ± 0.05
paracetamol	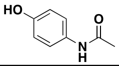	2.90	0.84 ± 0.05

Figure S9: a) Typical chromatograms for the IAM calibration mixture generated using method 1 with UV detection at 254 nm. b) Table of IAM calibration mixture components, their literature CHI(IAM)[S5] and average t_R±SD from n=8 repeats.

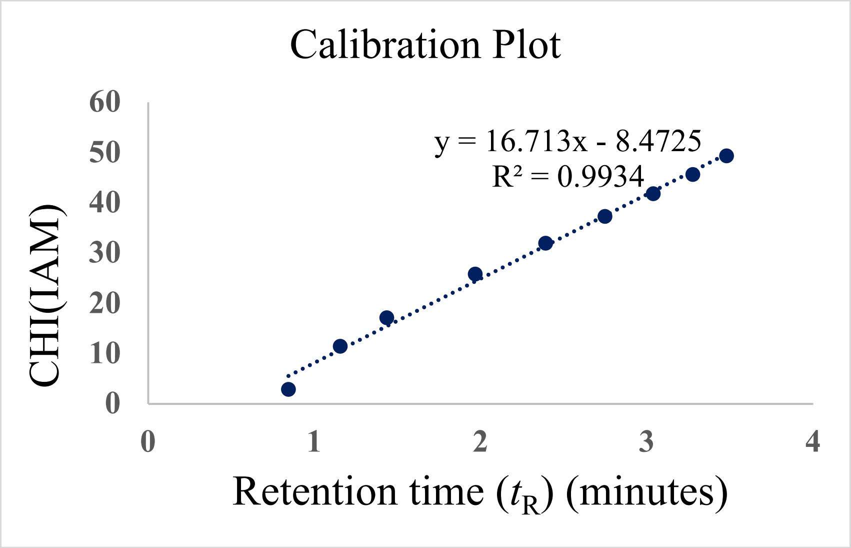

Figure S10: Calibration plot showing literature CHI (IAM)[S5] values against the t_R measured for each component in the IAM calibration mixture. The data is fitted with a straight-line y = 16.713x - 8.4725, correlation coefficient (r^2) $>$ 0.99.

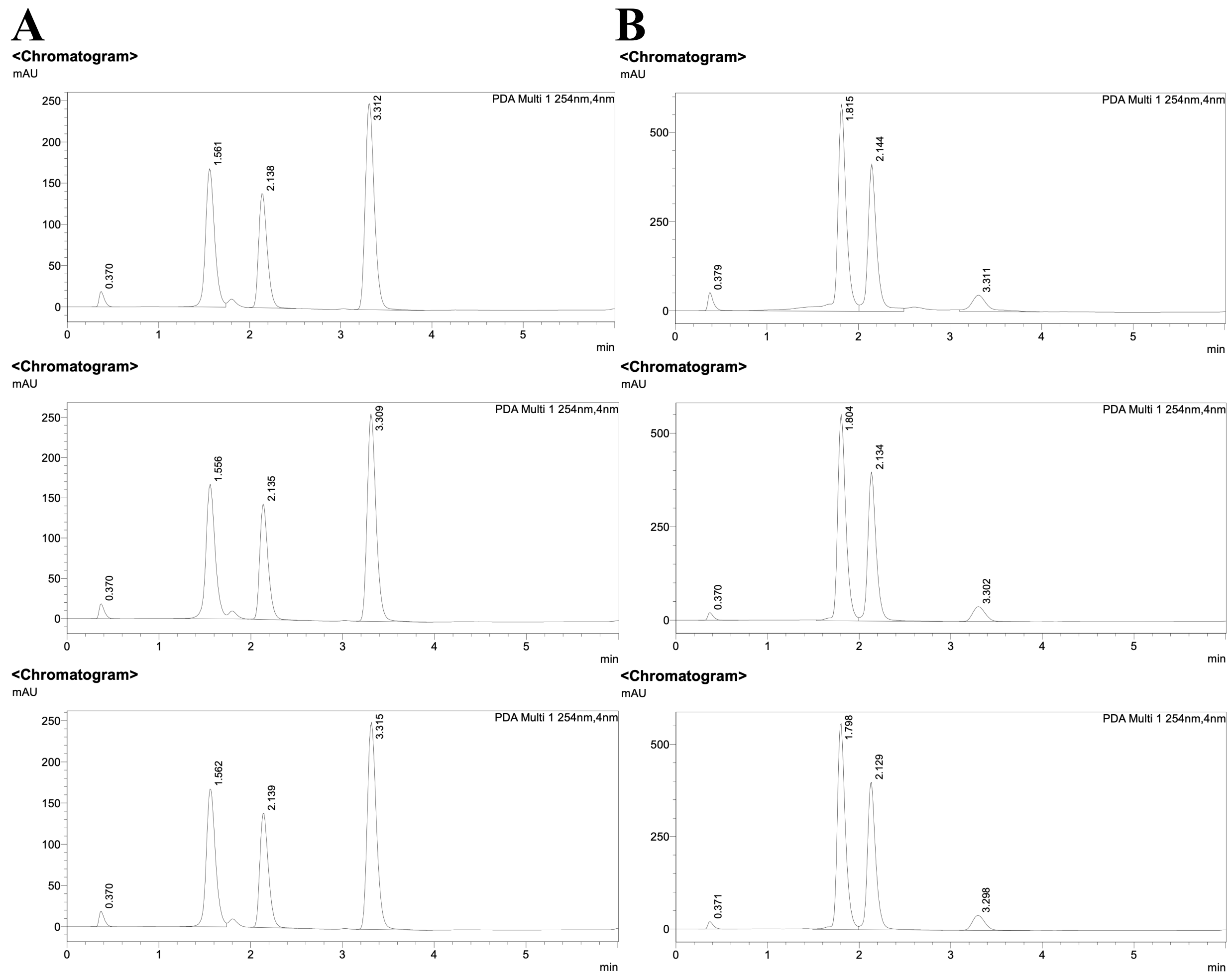

Figure S11: a) Typical chromatograms for suitability Test mixture 1 (nicardipine, warfarin and carbamazepine) and b) 2 (propanolol, indomethacin and colchicine) generated using method 1 with UV detection at 254 nm. All suitability tests were carried out daily in triplicate and the data summarized in Table S4.

A

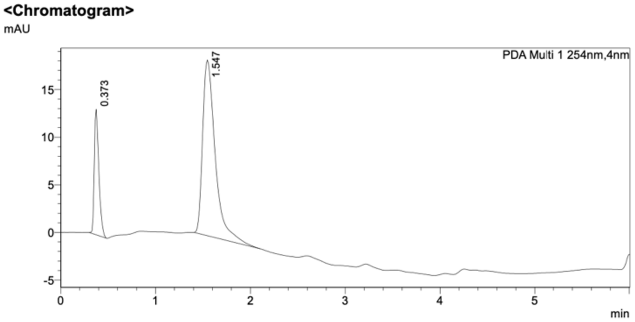

B

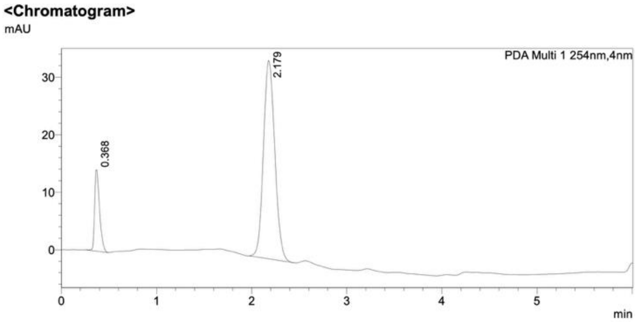

C

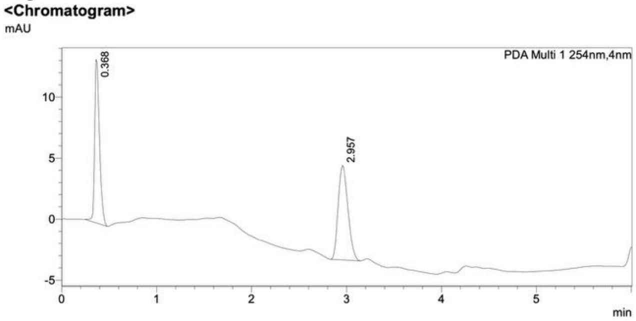

D

Molecule	t_R ± SD (mins) n=6	CHI(IAM)
Morphine	1.55 ± 0.03	17
Naloxone	2.18 ± 0.05	28
Fentanyl	2.96 ± 0.05	41

Figure S12: Typical chromatograms for a) morphine; b) naloxone; c) fentanyl generated using method 1 with UV detection at 254 nm. d) The mean t_R±SD of each molecule from n=6 repeats and the corresponding CHI(IAM) determined using calibration plot (Figure S9) is shown.

References

(S1) Wang, R.; Fu, Y.; Lai, L. A New Atom-Additive Method for Calculating Partition Coefficients. *J. Chem. Inf. Comput. Sci.* **1997**, *37*, 615–621.

(S2) Alexander, S. P. H.; Christopoulos, A.; Davenport, A. P.; Kelly, E.; Mathie, A. A.; Peters, J. A.; Veale, E. L.; Armstrong, J. F.; Faccenda, E.; Harding, S. D.; Davies, J. A.; Abbracchio, M. P.; Abraham, G.; Agoulnik, A.; Alexander, W.; Al-hosaini, K.; Bäck, M.; Baker, J. G.; Barnes, N. M.; Bathgate, R.; Beaulieu, J.-M.; Beck-Sickinger, A. G.; Behrens, M.; Bernstein, K. E.; Bettler, B.; Birdsall, N. J. M.; Blaho, V.; Boulay, F.; Bousquet, C.; Bräuner-Osborne, H.; Burnstock, G.; Caló, G.; Castaño, J. P.; Catt, K. J.; Ceruti, S.; Chazot, P.; Chiang, N.; Chini, B.; Chun, J.; Cianciulli, A.; Civelli, O.; Clapp, L. H.; Couture, R.; Cox, H. M.; Csaba, Z.; Dahlgren, C.; Dent, G.; Douglas, S. D.; Dournaud, P.; Eguchi, S.; Escher, E.; Filardo, E. J.; Fong, T.; Fumagalli, M.; Gainetdinov, R. R.; Garelja, M. L.; de Gasparo, M.; Gerard, C.; Gershengorn, M.; Gobeil, F.; Goodfriend, T. L.; Goudet, C.; Grätz, L.; Gregory, K. J.; Gundlach, A. L.; Hamann, J.; Hanson, J.; Hauger, R. L.; Hay, D. L.; Heinemann, A.; Herr, D.; Hollenberg, M. D.; Holliday, N. D.; Horiuchi, M.; Hoyer, D.; Hunyady, L.; Husain, A.; IJzerman, A. P.; Inagami, T.; Jacobson, K. A.; Jensen, R. T.; Jockers, R.; Jonnalagadda, D.; Karnik, S.; Kaupmann, K.; Kemp, J.; Kennedy, C.; Kihara, Y.; Kitazawa, T.; Kozielewicz, P.; Kreienkamp, H.-J.; Kukkonen, J. P.; Langenhan, T.; Larhammar, D.; Leach, K.; Lecca, D.; Lee, J. D.; Leeman, S. E.; Leprince, J.; Li, X. X. et al. The Concise Guide to PHARMACOLOGY 2023/24: G Protein-Coupled Receptors. *Br. J. Pharmacol.* **2023**, *180*, S23–S144.

(S3) Willighagen, E. L.; Mayfield, J. W.; Alvarsson, J.; Berg, A.; Carlsson, L.; Jeliazkova, N.; Kuhn, S.; Pluskal, T.; Rojas-Chertó, M.; Spjuth, O.; Torrance, G.; Evelo, C. T.; Guha, R.; Steinbeck, C. The Chemistry Development Kit (CDK) v2.0:

Atom Typing, Depiction, Molecular Formulas, and Substructure Searching. *J. Cheminform.* **2017**, *9*, 33.

(S4) Zhang, S.; Thompson, J. P.; Xia, J.; Bogetti, A. T.; York, F.; Skillman, A. G.; Chong, L. T.; LeBard, D. N. Mechanistic Insights into Passive Membrane Permeability of Drug-like Molecules from a Weighted Ensemble of Trajectories. *J. Chem. Inf. Model.* **2022**, *62*, 1891–1904.

(S5) Valko, K.; Du, C. M.; Bevan, C. D.; Reynolds, D. P.; Abraham, M. H. Rapid-Gradient HPLC Method for Measuring Drug Interactions with Immobilized Artificial Membrane: Comparison with Other Lipophilicity Measures. *J. Pharm. Sci.* **2000**, *89*, 1085–1096.
