# Supplementary material for: The Plasma Membrane may Serve as a Drug Depot to Drive the Extreme Potency of Fentanyl
Source: bioRxiv. 2025 Jul 26:2025.07.22.666146. Preprint. [Version 1] doi: 10.1101/2025.07.22.666146 (PMC12330492; doi:10.1101/2025.07.22.666146)
Supplement: Supplementary file 1 [file media-1.pdf]

Supplementary Materials for
The Plasma Membrane may Serve as a Drug Depot to Drive the Extreme Potency of Fentanyl

Joseph Clayton, George J Farmer, Jackie Glenn, Shailesh N Mistry, J Robert Lane, Meritxell Canals, Lei Shi, Lidiya Stavitskaya, Jana Shen*

*Corresponding author. Email: jana.shen@rx.umaryland.edu

This PDF file includes:

Materials and Methods

Figures S1 to S3

Tables S1 to S7

Other Supplementary Materials for this manuscript:

Movies S1 to S2

Data S1 to S2

Materials and Methods

Weighted-ensemble PME-CpHMD simulations

CpHMD parameterization. The all-atom particle mesh Ewald continuous constant pH molecular dynamics (PME-CpHMD) method (*9, 10*) in Amber 2024 (*18*) was used for the membrane permeation simulations. In brief, each titratable site (either in a protein or a small molecule) is represented by a fictitious particle λ which is bound between 0 (protonated) and 1 (deprotonated) through an internal variable θ, $\lambda = sin^2(\theta)$, which is propagated simultaneously with the atomic coordinates. The CpHMD simulations necessitate two types of parameters: the pK_a of the model titratable site in solution (i.e. model compound) and the potential mean force (PMF) function of λ for the model compound. For fentanyl, morphine, and naloxone, the model pK_a's were 8.4 (*33*), 8.2 (*35*), and 7.9 (*36*), respectively. As the pK_a of isotonitazene has not been experimentally determined, it was set to that of the structurally similar dimethytryptamine, 8.7 (*34*). The parameters in the PMF functions were determined through thermodynamic integration (TI) simulations in water as described below. More details are given in the original CpHMD development work (*9, 10*) and a recent tutorial (*37*).

The opioids were represented by the CGenFF force field (*38, 39*). Water molecules were represented by the modified TIP3P model (*40, 41*). The force field parameters of sodium and chloride ions were taken from Refs (*42, 43*). For both TI and solution titration simulations for validation of the solution pK_a values of the opioids, a solvated system was built by solvating the opioid using a water box with a distance of at least 15 Å between the nearest water oxygen and the heavy atom on the small molecule. Sodium and chloride ions were added to reach an ionic strength of 0.15 M, with one additional chloride ion added to compensate for the net charge of 1 at pH 7.5. During the CpHMD titration, the effect of net charge was accounted for using a uniform background charge (plasma) in the PME correction term for propagating atomic coordinates (*9, 10, 44*). Each system was minimized for 5,000 steps, then seven independent replicas were created by fixing θ_i (0.2, 0.4, 0.6, 0.7854, 1.0, 1.2, or 1.4). Each replica was heated to 300 K under constant volume over 50 ps with a 5 kcal/mol/Å restraint on the heavy opioid atoms, then restraints were gradually relaxed over 100 ps under constant pressure of 1 bar. Pressure was controlled using the Monte-carlo barostat (*45*) while temperature was controlled at 300 K using Langevin dynamics with a collision frequency

of 1 ps^{-1}. Long-range electrostatic interactions were calculated using particle mesh Ewald (PME) method with a real-space cutoff of 12 Å and a 1 Å grid spacing for the reciprocal space calculations. Lennard-Jones energies and forces were smoothly switched off over the range of 10 to 12 Å. The mean force $\langle \partial U/\partial \theta \rangle$ was then calculated over a 10-ns simulation for each θ; fitting the calculated mean force at a series of θ values to the partial derivative of the quadratic PMF function yields the two parameters in the PMF (*37*). Note that, in order to minimize fitting errors, fitting was done in the θ space and not the transformed λ (*37*). Following the TI simulations, titration simulations were performed to verify the experimental pK_a's in solution are recapitulated by CpHMD simulations. Six separate 20 ns simulations were conducted at different pH conditions, ranging from 7.0 to 9.5. To verify the parameters obtained from TI, the average deprotonation fraction over the final 10 ns of each simulation was measured then used to fit to the generalized Henderson-Hasselbach equation to obtain a calculated pK_aFigure S2.

System preparation for the WE-CpHMD simulations. To prepare the initial system, CHARMM-GUI (*46*) was used to create a bilayer with a 5:5:1 ratio of POPC, POPE, and cholesterol; this composition was chosen to mimic mammalian neural soma (*47*) and led to 66 lipids total (33 lipids per leaflet). An opioid was then induced and placed 30 Å from the center of the bilayer. A water layer of 22.5 Å was added above and below the bilayer. Sodium and chloride ions were added to reach an ionic strength of 0.15 M, and an additional chloride ion was added to neutralize the system at pH 7.5. The system was minimized for 5000 steps, then heated under constant volume to 300 K over 125 ps with restraints on lipid positions and dihedrals. These restraints were then gradually removed over 2.25 ns under constant pressure of 1 bar, followed by a 50 ns simulation to equilibrate the membrane. All steps were done using Amber24 (*18*). The CHARMM36 lipid force field (*48*) was used. All other parameters and settings were identical to those described above for the solution CpHMD simulations of opioids.

The weighted-ensemble (WE) protocol. WE simulations, in brief, involve iteratively evolving a number of independent replicate systems called walkers. After each iteration a progress coordinate (or several coordinates) is calculated and used to place each walker into predefined bins; within each occupied bin, walkers are either replicated or removed such that the number of walkers per bin

meets a target value. By updating a statistical weight for each walker after each split (replication) or merge (removal) both equilibrium and kinetic information can be estimated.

The progress coordinate used was defined as the z-position of the center of mass of the opioid (excluding hydrogen atoms), with the center of the membrane (defined by the center of all phosphorous atoms of POPC and POPE lipids) as the origin. Bin boundaries were set based on the environment: when the opioid was in solvent ($|z| > 20$ Å) boundaries were set 5 Å apart, while when inside the membrane (-20Å $< z <$ 20Å) boundaries were set 0.5 Å apart. This defined 85 bins between $z = -25$ and $z = 55$. A steady state WE simulation was prepared, where a walker would be recycled if $z > 55$ Å or $z < -25$ Å at the end of the iteration. This was done to ensure the opioid does not cross the periodic boundary and that membrane partitioning occurs in one direction.

The WE simulations were conducted using WESTPA 2.0 (*12*) and Amber24 (*18*). The target count for each bin was set to 4; each iteration involved simulating each walker for 100 ps at pH 7.5 using all-atom PME-CpHMD (*10*). All analysis was done using the WESTPA package and MDTraj (*49*). The rate of permeation was analyzed by dividing the system into three region based on the z-position from the bilayer center: extracellular ($z > 20$ Å), bilayer ($-20 < z < 20$ Å), and intracellular ($z < -20$ Å). The probability flux from extracellular to intracellular $f_{ex \to in}$ was measured using the w_assign and w_direct tools within the WESTPA package (*12,50*), and then was used to calculate the effective permeability and mean first passage time (MFPT) following Zhang et al. (*13*):

$$\begin{aligned} P_m &= f_{ex \to in} * l_d \\ MFPT &= \frac{p_{ext}}{f_{ex \to in}} \end{aligned} \tag{S1}$$

In the above equations, l_d represents the depth of the effective reaction volume while p_{ext} represents the fraction of trajectories that most recently sampled the extracellular state versus the intracellular state. The effective reaction volume is the region where the surface of the membrane influences neighboring molecules, thus events in bulk solvent (such as stirring) do not affect molecules in this region; following Zhang et al. (*13*), we set this to half of the height of the overall water buffer (l_d = 22.5 Å) in our analysis. Since the intracellular region was set as a recycle

condition, there are no trajectories that sample the intracellular before sampling the extracellular– thus $p_{ext} = 1$.

The rate of deprotonation within the bilayer was estimated in a similar manner as described above. First, fentanyl was divided along two dimensions: the progress coordinate z-position (to determine if fentanyl was within the bilayer) and λ (to determine whether the amine is protonated or deprotonated). The bilayer region was defined as described above; the protonated state was defined as $\lambda < 0.2$ and the deprotonated state as $\lambda > 0.8$.

Bioluminescence resonance energy transfer (BRET) experiments

Materials DMEM was from Sigma-Aldrich, FBS was from Sigma-Aldrich, PEI was from Poly-Sciences Inc, poly-D-lysine was from Fisher Scientific, coelenterazine h was from NanoLight Technology (Prolume Ltd), morphine was from Tocris, fentanyl was from Sigma-Aldrich, DAMGO was from Hello Bio, Naloxone was from Hello Bio and D-PBS was from Gibco.

Cell culture and transfection Human embryonic kidney 293 T (HEK 293T) cells were cultured at 37 °C, 5% CO2 in Dulbecco's modified eagle medium (DMEM) supplemented with 10% (v/v) fetal bovine serum (FBS). For transfection, cells were plated in 10 cm Petri dishes (3 x 106 cells per dish) and allowed to grow overnight in full media at 37 °C, 5% CO_2. 24h later, cells were transiently transfected, using a 1:6 total DNA to PEI ratio and the following DNA constructs: 2 μM of Gαi2, 1 µM of Gβ1-Venus(156-239), 1 µM of Gγ2-Venus(1-155), 1 µM of masGRK3ct-Rluc8 and 1 μM of MOR [SNAPmMOR]. DNA/PEI mixtures were added to the cells. 24 post-transfection, cells were plated in Greiner poly-D-lysine-coated, white bottom 96-well plates (SLS) in full media.

BRET measurements. On the day of the assay (48h post-transfection), cells were washed once with D-PBS (Lonza, SLS) and incubated in D-PBS for 30 min at 37 °C. The Rluc substrate coelenterazine h was added to each well (final concentration of 5 μM) and treated as specified below. BRET measurements (Venus and Rluc emission signals at 535 and 475 nm, respectively), were performed using a PHERAstar FSX microplate reader (BMG Labtech) at 37 °C. BRET ratio was calculated as the emission at 535 nm divided by the emission intensity at 475 nm signal and corrected for the vehicle BRET ratio signal.

Kinetic BRET experiments. After a 5 min baseline read after the addition of coelenterazine h (final concentration of 5 µM), morphine (1 μM), fentanyl (100 nM) or vehicle were added, and the signal read for 10 min. Then, 100 nM of naloxone or vehicle were injected to the wells. At this concentration, naloxone was demonstrated to partially reverse the G protein activation evoked by morphine (1 μM) and fentanyl (100 nM) to similar levels. The BRET signal was read for further 15 min prior to 3 washouts of the cells with D-PBS, re-addition of coelenterazine h (final concentration of 5 μM) in drug-free D-PBS and measurement of the BRET signal for further 20 min until the final addition of 10 µM naloxone to fully reverse MOR induced G protein activation.

Ligand-release BRET sensor experiments. Untransfected HEK293T cells were incubated with vehicle or 10 µM of fentanyl, DAMGO or morphine for 30 min at 37°C. After incubation, the supernatant of the cells was recovered (no wash) and cells washed, 5x with D-PBS and supernatants recovered (wash 1, 2,3,4, 5, respectively). 100 µL of these supernatants were then used to stimulate a plate containing cells transfected with MOR, and the G protein activation sensor constructs (Gαi2, Gβ1-Venus(156-239), Gγ2-Venus(1-155) and masGRK3ct-Rluc8). Direct application of vehicle or 10 μM of fentanyl, DAMGO or morphine was used as a control. BRET signal was measured as above after 10 min incubation.

Immobilized affinity membrane (IAM) chromatography experiment

Equipment and methods. Immobilised artificial membrane-high performance liquid chromatography (IAM-HPLC) was performed using a conventional HPLC set up fitted with a IAM P.C DD2 column (30 x 4.6 mm, 10 µm, 300 Å) (Regis technologies Inc, Chicago, USA). The column was maintained at 30°C with a flow rate of 1.5 mL/min and UV detection at 254 nm. The system consisted of a Shimadzu systems controller SCL-40, degassing unit DGU-405, solvent delivery module LC-40D XR, auto sampler SIL-40C XR, column oven CTO-40C and a photo diode array (PDA) detector SPD-M40 (Shimadzu, Kyoto, JPN). Solvent A contained ammonium acetate (50 mM) (Sigma-Aldrich, Gillingham, UK) in Milli-Q water at pH 7.4 and solvent B was acetonitrile (Thermo-Fisher Scientific, Loughborough, UK).

Method 1: 0-85% B over 4.75 min; 85% B for 2 min; 85–0% B over 0.5 min, then 0% B. All standards and samples were dissolved via dropwise addition of DMSO, before being diluted in water to 1 mM final concentration. Each sample was loaded in 10 µL injections and repeated

in triplicate on 2 separate days. IAM calibration mixture (Bio Mimetic Chromatography Ltd, Stevenage, UK) composition: octanophenone, heptanophenone, hexanophenone, valerophenone, butyrophenone, propiophenone, acetophenone, acetanilide, paracetamol. Test mixture 1: propranolol, indomethacin and colchicine; Test mixture 2: warfarin, carbamazepine, nicardipine. Samples: fentanyl, buprenorphine, naloxone, [D-Ala^2, N-MePhe^4, Gly-ol]-enkephalin (DAMGO), morphine - all commercially available from standard suppliers.

Calibration plot. The system was calibrated by introducing the IAM calibration mixture (10 µL)using method 1 and plotting the t_R of each component in the mixture against chromatographic hydrophobicity index (CHI (IAM)) values (*51*) from the literature. Typical chromatograms, retention times (t_R) and values of CHI (IAM) from the literature are shown in Figure S9. A calibration plot and equation for the line of best fit was generated in Microsoft Excel (version 16.88) and the Pearson correlation coefficient (r) was calculated to be >0.99, as summarized in graphical plot (Figure S10).

Column performance and suitability test. Before samples were analysed, an assessment of column performance was carried out daily by introducing using Test mixtures 1 and 2 (Figure S11) using Method 1. The retention time of the components in each mixture were converted to CHI(IAM) values using the calibration plot in (Figure S10). The column was deemed suitable for analysis if all measured CHI (IAM) values were within ± 5 of their corresponding literature values (*52*) Examples of typical chromatograms are shown in (Figure S11) and all data is summarised in Table S4.

Sample testing. Samples were analysed using method 1 and typical chromatograms are shown in (Figure S12). Retention time was converted to CHI (IAM) values for each sample using the calibration plot (Figure S10).

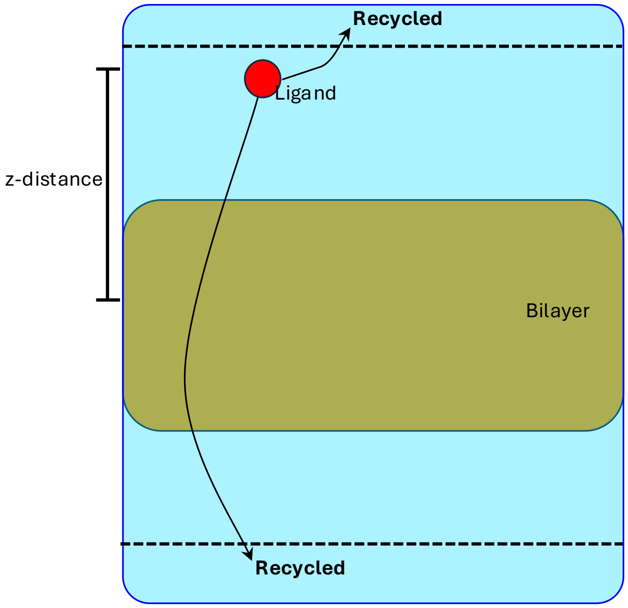

Figure S1: **Schematic of the steady-state condition.** Schematic of the steady-state condition used for the WE-CpHMD simulation. Once the ligand either moves far away from the membrane or permeates through, the walker is recycled–meaning the walker is terminated and a new one is started from the initial state.

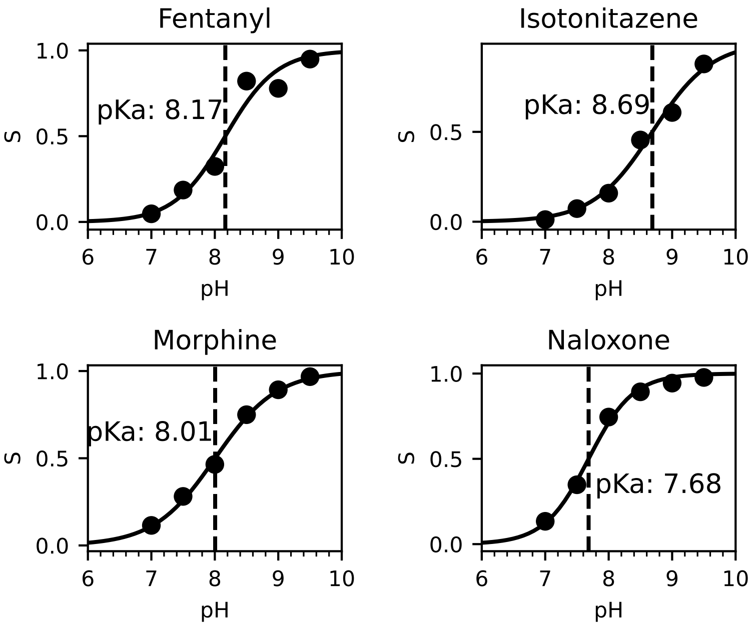

Figure S2: **Simulated solution titration curves of the four studied compounds.** Deprotonation fraction at different simulation pH conditions. 20 ns simulations were conducted at six different pH conditions ranging from 7.0 to 9.5. The final 10 ns of each was then used to calculate the deprotonation fraction. The calculated pK_a's are given.

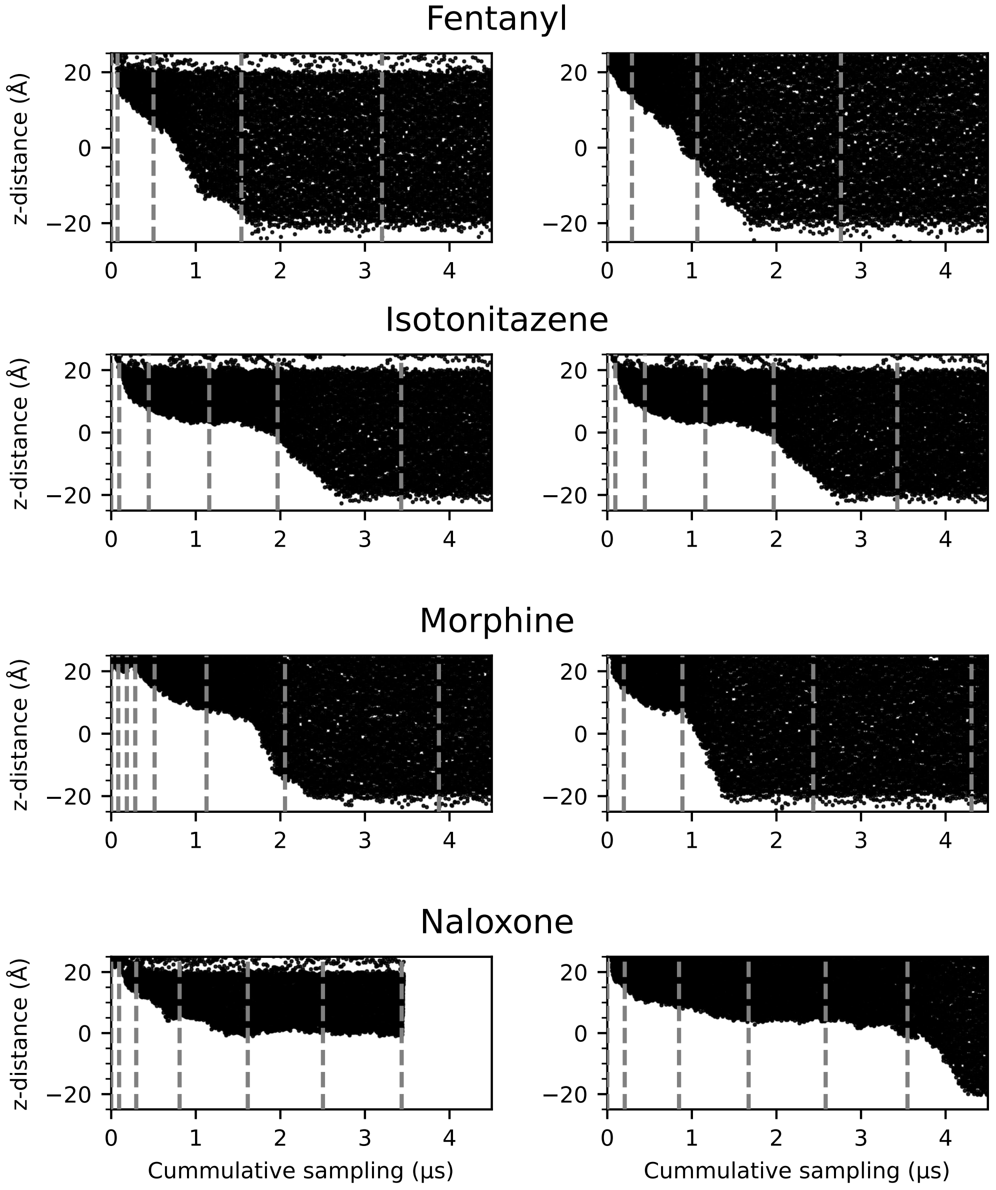

Figure S3: **Fentanyl requires the least simulation time to permeate the membrane.** z-position vs. cumulative simulation time of fentanyl, morphine, isotonitazene, and naloxone from the first (left) and second (right) sets of WE-CpHMD simulations. Gray dashed lines are drawn at every 50 WE iterations; for simplicity, only the first 4.5μs is shown.

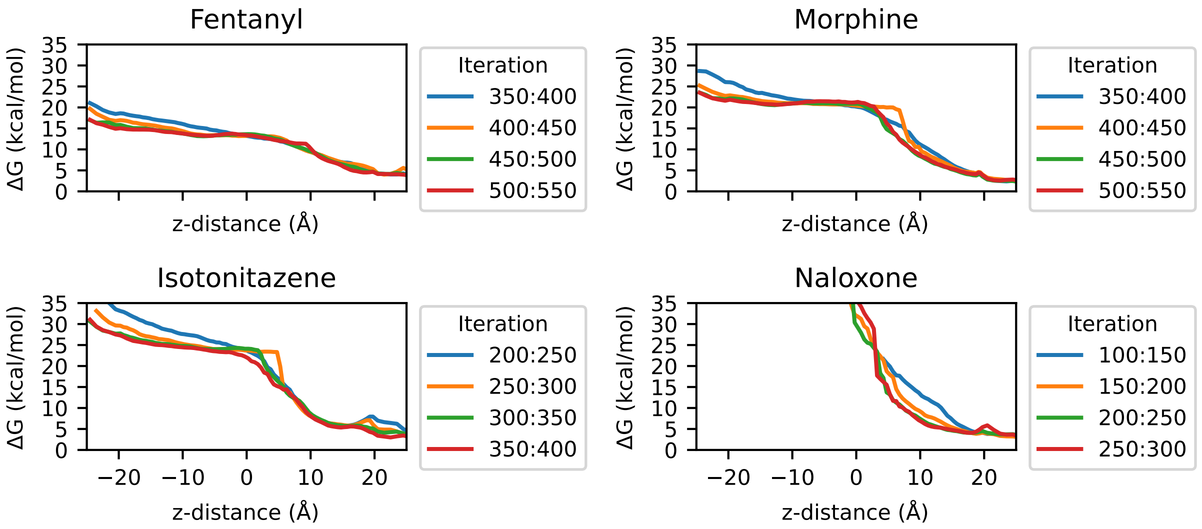

Figure S4: **Convergence of the potential of mean force calculations (first trial).** Convergence of the potential mean force, calculated using four windows containing 50 iterations, for the first set of weighted ensemble simulations.

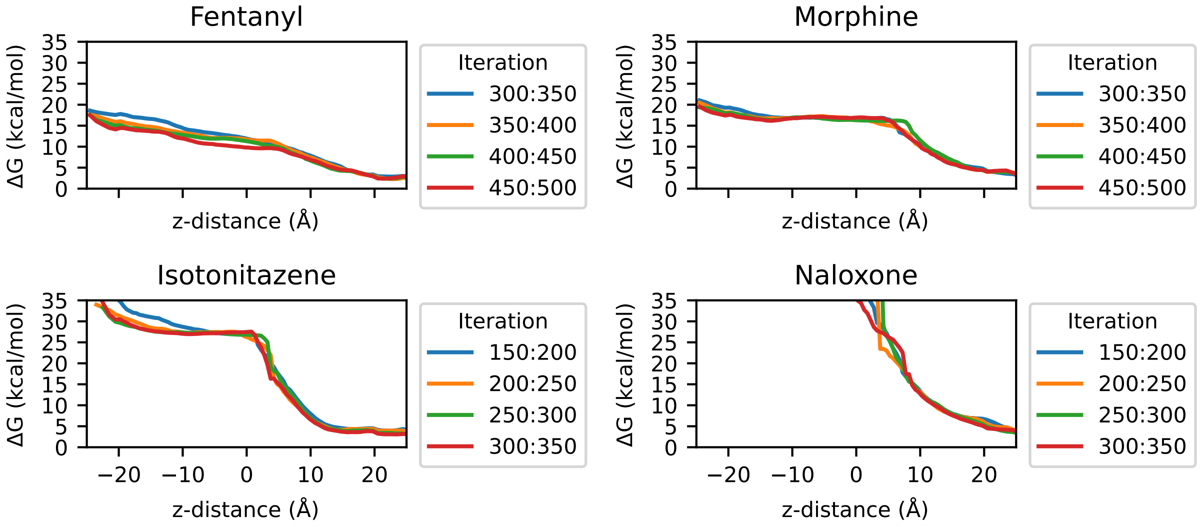

Figure S5: **Convergence of the potential of mean force calculations (second trial).** Convergence of the potential mean force, calculated using four windows containing 50 iterations, for the first set of weighted ensemble simulations.

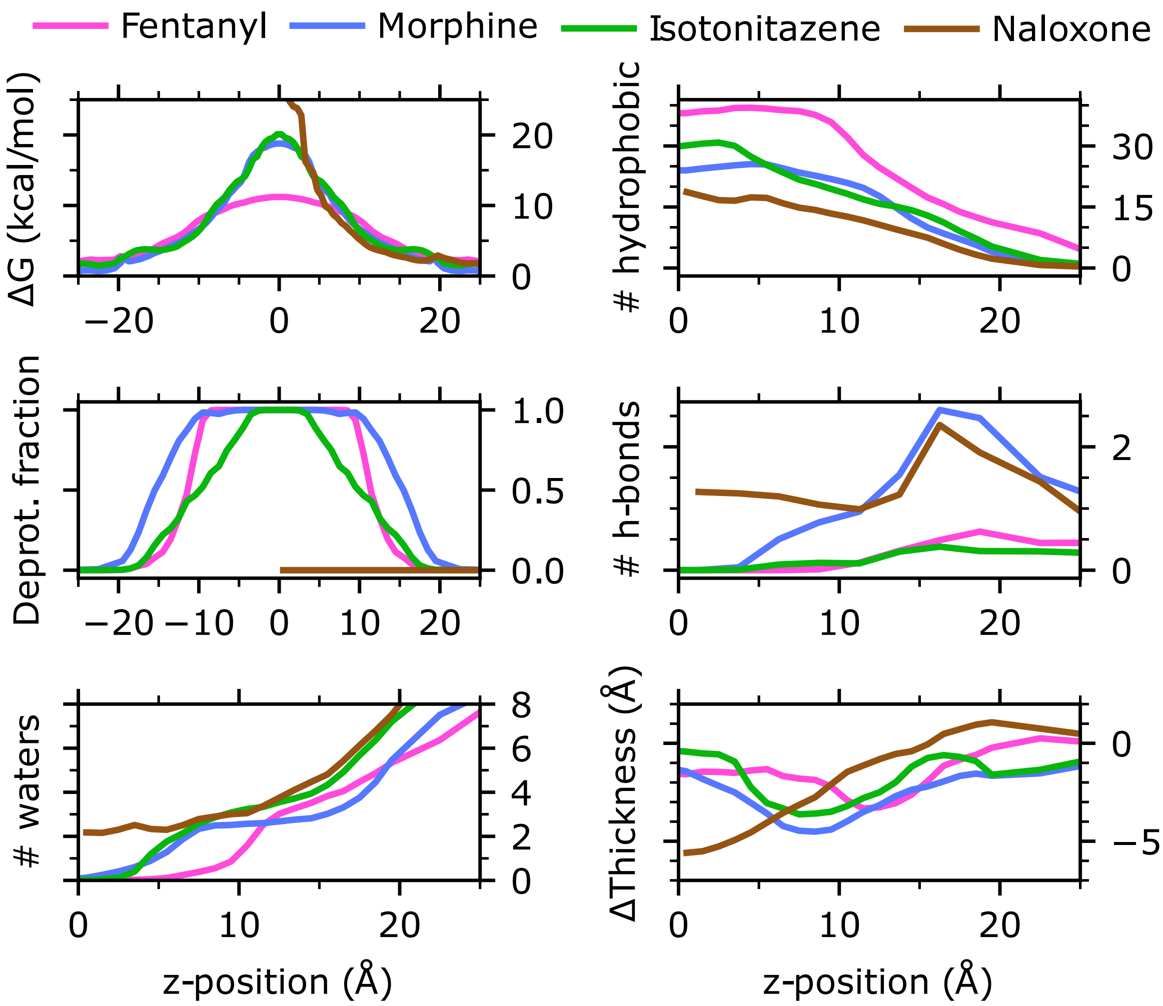

Figure S6: **Trends in permeation events are reproducible.** Characterization of the membrane permeation properties of fentanyl, morphine, isotonitazene, and naloxone from the simulation set 1. **Left.** Free energy profile (top), deprotonation fraction (middle) and the number of water within 3.4 Å from any heavy atom of the permeant (bottom) as a function of its *z*-position. The profiles are symmetrized about $z = 0$ following Ref (*13*). **Right.** Number of hydrophobic contacts (top) and h-bonds (middle) between the permeant and lipid molecules and the change of the membrane local thickness around the permeant (bottom) as a function of *z*. cylinder around the permeant COM. The average value of the local thickness when the permeant COM is > 30 Å from the membrane is used as a reference. Data are taken from the final 100 iterations of the first set of WE-CpHMD simulations.

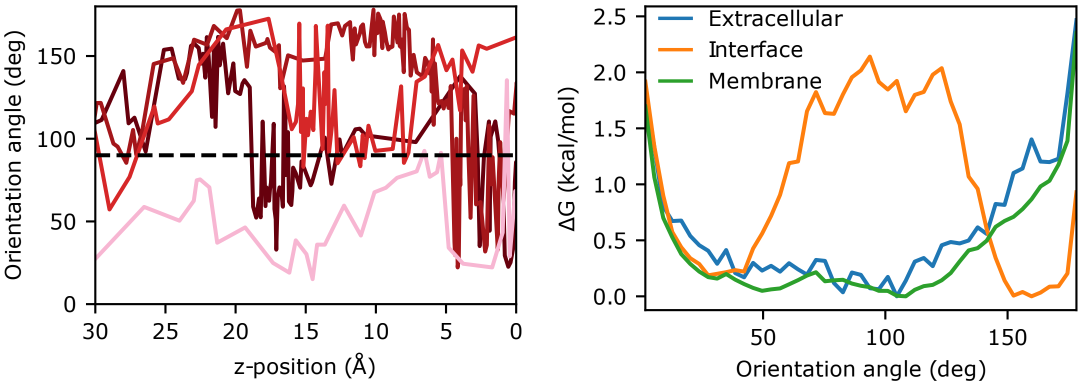

Figure S7: **Fentanyl prefers a vertical orientation at the interface in simulation set 1. Left.** Fentanyl orientation angle along stitched histories of walkers reaching the center of the membrane. **Right.** Potential mean force along the orientation angle while fentanyl is in the extracellular region, at the membrane interface, and within the membrane.

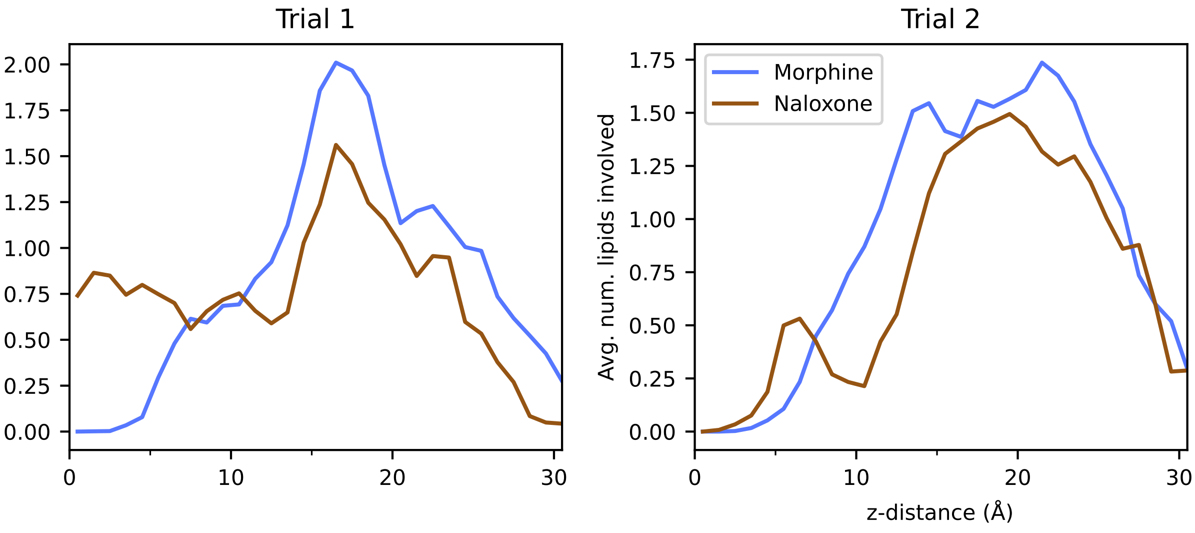

Figure S8: **Morphine and naloxone can interact with two separate phospholipid molecules while at the membrane interface.** Average number of lipids involved in hydrogen bonds with either morphine or naloxone, as a function of z-distance.

A

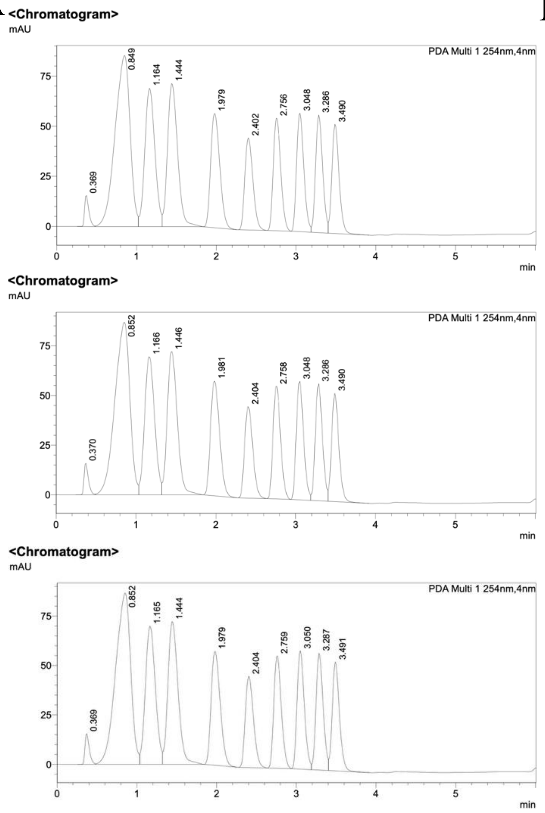

B

Calibration Standards	Structure	CHI (IAM)[54]	t_R ± SD (mins) n=8
octanophenone	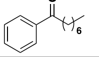	49.4	3.48 ± 0.04
heptanophenone	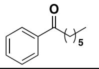	45.7	3.28 ± 0.04
hexanophenone	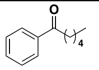	41.8	3.04 ± 0.04
valerophenone	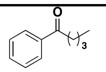	37.3	2.75 ± 0.04
butyrophenone	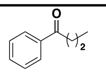	32.0	2.39 ± 0.04
propiophenone	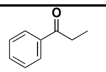	25.9	1.97 ± 0.04
acetophenone	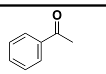	17.2	1.44 ± 0.04
acetanilide	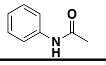	11.5	1.16 ± 0.05
paracetamol	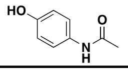	2.90	0.84 ± 0.05

Figure S9: a) Typical chromatograms for the IAM calibration mixture generated using method 1 with UV detection at 254 nm. b) Table of IAM calibration mixture components, their literature CHI(IAM) (*51*) and average t_R±SD from n=8 repeats.

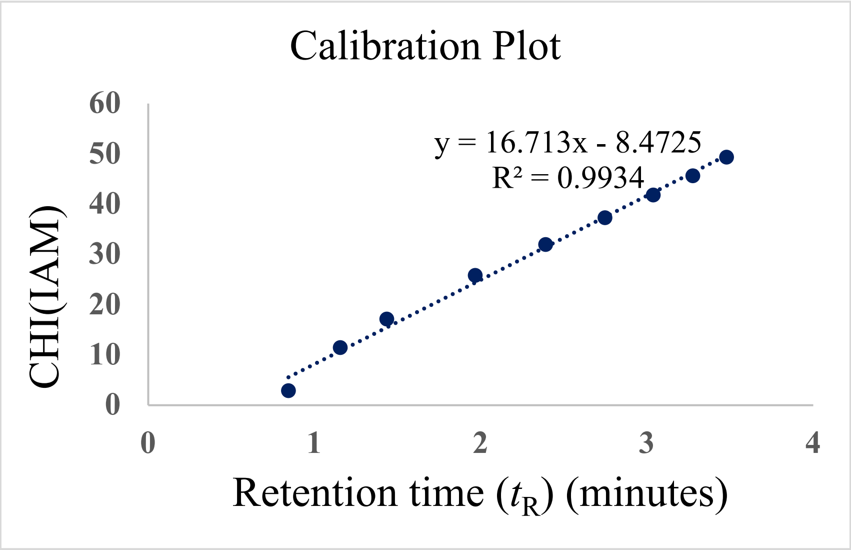

Figure S10: Calibration plot showing literature CHI (IAM) (*51*) values against the t_R measured for each component in the IAM calibration mixture. The data is fitted with a straight-line y = 16.713x - 8.4725, correlation coefficient (r^2) > 0.99.

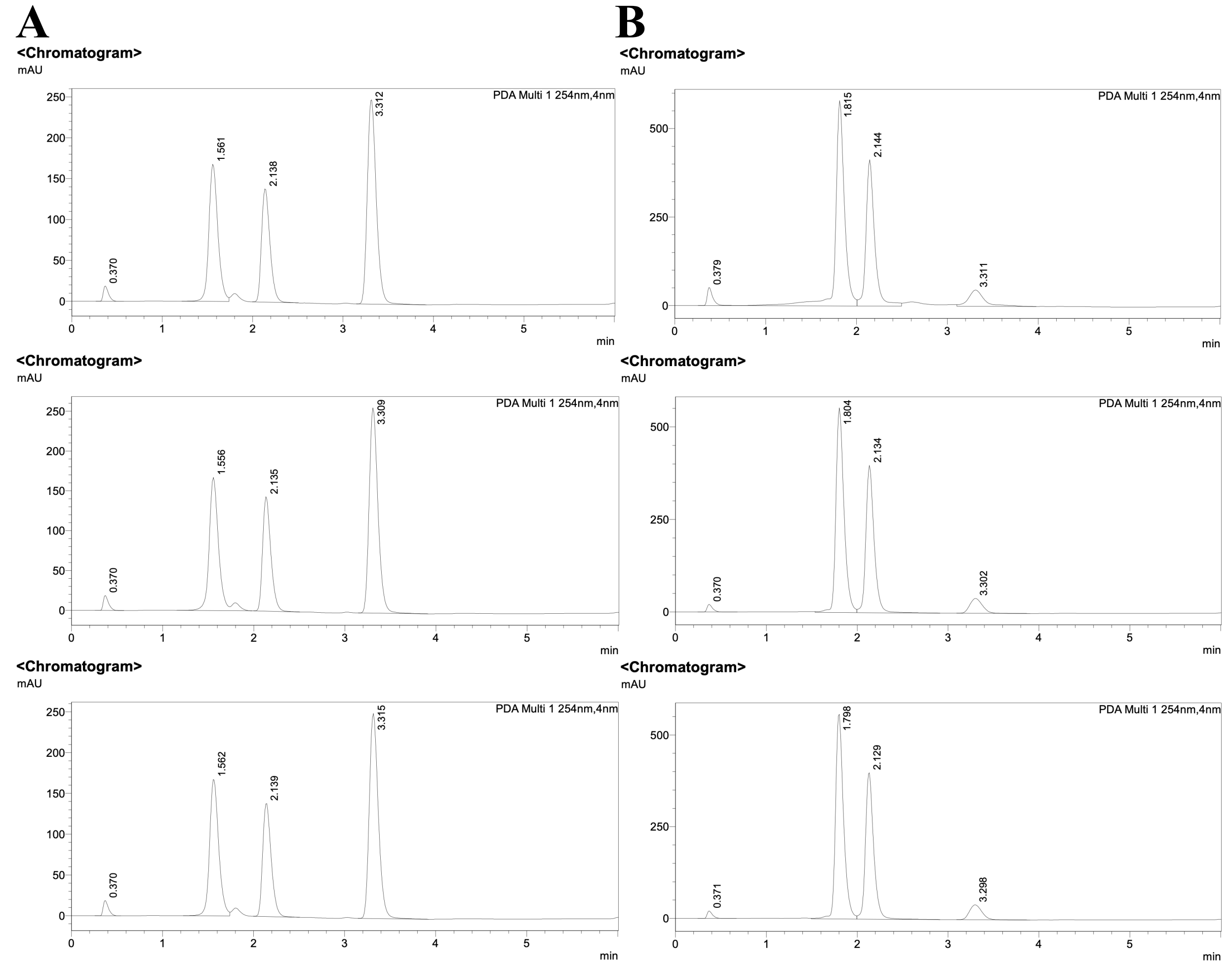

Figure S11: a) Typical chromatograms for suitability Test mixture 1 (nicardipine, warfarin and carbamazepine) and b) 2 (propanolol, indomethacin and colchicine) generated using method 1 with UV detection at 254 nm. All suitability tests were carried out daily in triplicate and the data summarized in Table S4.

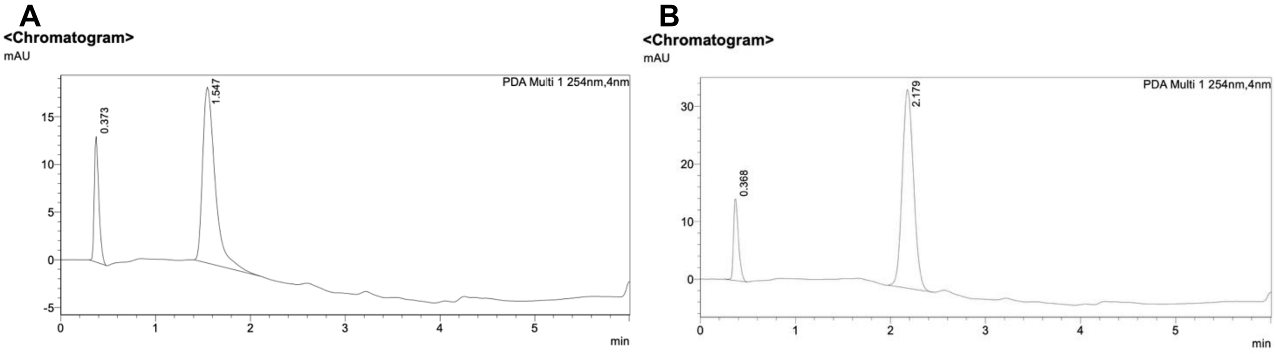

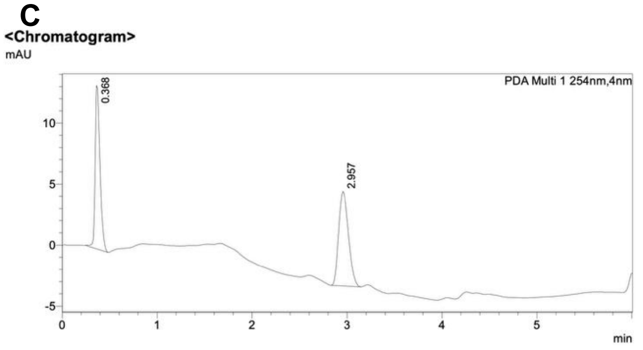

D

Molecule	t_R ± SD (mins) n=6	CHI(IAM)
Morphine	1.55 ± 0.03	17
Naloxone	2.18 ± 0.05	28
Fentanyl	2.96 ± 0.05	41

Figure S12: Typical chromatograms for a) morphine; b) naloxone; c) fentanyl generated using method 1 with UV detection at 254 nm. d) The mean t_R±SD of each molecule from n=6 repeats and the corresponding CHI(IAM) determined using calibration plot (Figure S9) is shown.

Table S1: Parameters in the model titration PMF for the four compounds

Molecule	A	B
Fentanyl	-58.059565	0.100857
Morphine	-37.405443	0.136572
Isotonitazene	-51.327454	0.325572
Naloxone	-74.288134	0.046556

Table S2: Number of iterations and cumulative sampling time (µs) for each WE-CpHMD simulation

Molecule	First trial		Second trial	
	Iteration	Time	Iteration	Time
Fentanyl	550	14.9	500	15.7
Morphine	550	11.3	500	15.5
Isotonitazene	400	8.5	350	9.5
Naloxone	300	3.4	350	7.0

Table S3: Physicochemical properties of four studied compounds

Molecule	Mol weight	Rot. bonds	H-bond donor	H-bond acceptor	xLogP
Fentanyl	336	6	0	3	3.94
Isotonitazene	410	9	0	4	3.62
Morphine	285	0	2	2	0.49
Naloxone	327	2	3	2	0.31

Molecular weight is unit of g/mol. xlogP (calculated octanol-water partition coefficient) (*53*) values are taken from the Database IUPAC/BPS Guide to Pharmacology (*54*) which reports the CDK calculations (*55*).

Table S4: Hydrophobicity index from immobilized affinity membrane (IAM) experiments

Name	Acid/base profile	t_R± SD (mins,n=6)	Measured CHI (IAM)	Reference CHI (IAM)
Propanolol	basic	3.30 ± 0.04	47	42
Nicardipine	basic	3.31 ± 0.03	47	45
Indomethacin	acidic	2.13 ± 0.06	27	30
Warfarin	acidic	1.56 ± 0.06	18	20
Carbamazepine	neutral	2.14 ± 0.02	27	27
Colchicine	neutral	1.80 ± 0.07	22	23

Components of suitability Test Mixtures 1 and 2 and their corresponding acid/base profile. The average t_R±SD from n=6 repeats measured using method 1 was used to determine CHI(IAM) for each molecule using the calibration plot (Figure S10). The column was deemed suitable for analysis due to measured CHI(IAM) values being within ±5 of reference CHI(IAM) from the literature.
